# UVA induces retinal photoreceptor cell death via receptor interacting protein 3 kinase mediated necroptosis

**DOI:** 10.1038/s41420-022-01273-1

**Published:** 2022-12-12

**Authors:** Zhen Yu, Victor S. M. C. Correa, Nikolaos E. Efstathiou, Henar Albertos-Arranz, Xiaohong Chen, Kenji Ishihara, Yasuhiro Iesato, Toshio Narimatsu, Dimitrios Ntentakis, Demetrios G. Vavvas

**Affiliations:** 1 Shenzhen Eye Hospital, Jinan University, Shenzhen Eye Institute, Shenzhen, China; 2 Retina Service, Ines and Fred Yeatts Retina Research Laboratory, Angiogenesis Laboratory, Department of Ophthalmology, Massachusetts Eye and Ear, Harvard Medical School, Boston, MA USA; 3 Department of Physiology, Genetics and Microbiology, University of Alicante, Alicante, Spain

**Keywords:** Necroptosis, Retinal diseases

## Abstract

Ultraviolet light A (UVA) is the only UV light that reaches the retina and can cause indirect damage to DNA via absorption of photons by non-DNA chromophores. Previous studies demonstrate that UVA generates reactive oxygen species (ROS) and leads to programmed cell death. Programmed cell death (PCD) has been implicated in numerous ophthalmologic diseases. Here, we investigated receptor interacting protein 1 and 3 (RIPK1 and RIPK3) kinases, key signaling molecules of PCD, in UVA-induced photoreceptor injury using in vitro and ex vivo models. UVA irradiation activated RIPK3 but not RIPK1 and mediated necroptosis through MLKL that lie downstream of RIPK3 and induced apoptosis through increased oxidative stress. Moreover, RIPK3 but not RIPK1 inhibition suppresses UVA-induced cell death along with the downregulation of MLKL and attenuates the levels of oxidative stress and DNA fragmentation. In conclusion, these results identify RIPK3, not RIPK1, as a critical regulator of UVA-induced necroptosis cell death in photoreceptors and highlight RIPK3 potential as a neuroprotective target.

## Introduction

Human exposure to Ultraviolet (UV) radiation is increasing due to ozone depletion and global climate change on the Earth’s surface [1]. UV radiation contains more energy than visible or infrared light and therefore has more potential for biological damage. In fact, it is thought to be associated with many ocular diseases due to its direct irradiation to human eyes [2, 3]. In a study of human RPE cells (ARPE-19), a time dependent cell death induced by UVC (100–280 nm) is observed [4]. Another study demonstrates that UVB (280–315 nm) leads to the formation of DNA strand breaks in RPE cells, which is a risk factor for the development of age-related macular degeneration (AMD) [5–7]. However, the mechanism of UVA (315–400 nm) induced retinal cell damage and identification of potential new protection or treatment methods have been relatively neglected as an area of investigation.

UVA radiation is the only UV light that reaches the retina and causes indirect damage to DNA via absorption of photons by non-DNA chromophores [8, 9]. This generates reactive oxygen species (ROS) like singlet oxygen or hydrogen peroxide that oxidize DNA bases causing cellular DNA damage, which leads to programmed cell death (PCD) [10–12]. PCD is responsible for the pathogenesis of various anterior and posterior ocular diseases, primarily including Fuchs endothelial corneal dystrophy (FECD), glaucoma, neovascular AMD, and retinitis pigmentosa (RP) [13–18]. Numerous factors affect the PCD process, including altering protein levels and other macromolecular substance dysfunction, mitochondrial dysfunction, and oxidative stress (OS) [19]. Recent studies show that multiple cellular gene expression mediates PCD, which mainly includes apoptosis, autophagy, necroptosis, ferroptosis, and pyroptosis [20–24].

Necroptosis, as an alternative form of PCD, is also referred to as programmed necrosis [25]. Necroptosis displays morphological features similar to both necrosis and apoptosis and is tightly regulated by a multiprotein interaction [26]. Necroptosis can compensate for apoptosis after inhibition of Caspases [27–30]. Necroptosis depends on a signaling complex called necrosome, containing receptor-interacting protein kinase 1(RIPK1), RIPK3, and mixed-lineage kinase domain-like (MLKL) [31–33]. The interaction between RIPK1 and RIPK3, gives rise to RIPK3–RIPK3 homo-interaction and RIPK3 auto-phosphorylation, which forms the key elements of the necrosome. Mixed lineage kinase domain-like (MLKL) is recruited and phosphorylated only by RIPK3, then translocated to the cell membrane to execute necroptosis [34–37]. Although both RIPK3 and RIPK1 are required to induce necroptosis, RIPK3 alone can promote necroptosis when overexpressed in cells [38].

RIPK3 is not only a vital signaling factor in the necroptotic pathway but also has important effects on development, induction of pluripotent stem cells, general homeostasis, tissue damage response, and antiviral immunity [39–42]. A previous study demonstrated that RIP3 mediates photoreceptor injury/death in an experimental retinal detachment mouse model. [43]. Additionally, the knockdown or depletion of RIPK3 decreases photoreceptor cell death in zebrafish and mice [44, 45]. However, not much is known about UVA-induced photoreceptor damage via RIPK related pathways. Here, we aimed to investigate the effects of necroptosis and the role of RIPK1 and RIPK3 in an in vitro and ex vivo models of UVA-induced photoreceptor injury.

## Results

### UVA induces photoreceptor cells death and reduces cell viability

**Fig. 1:**
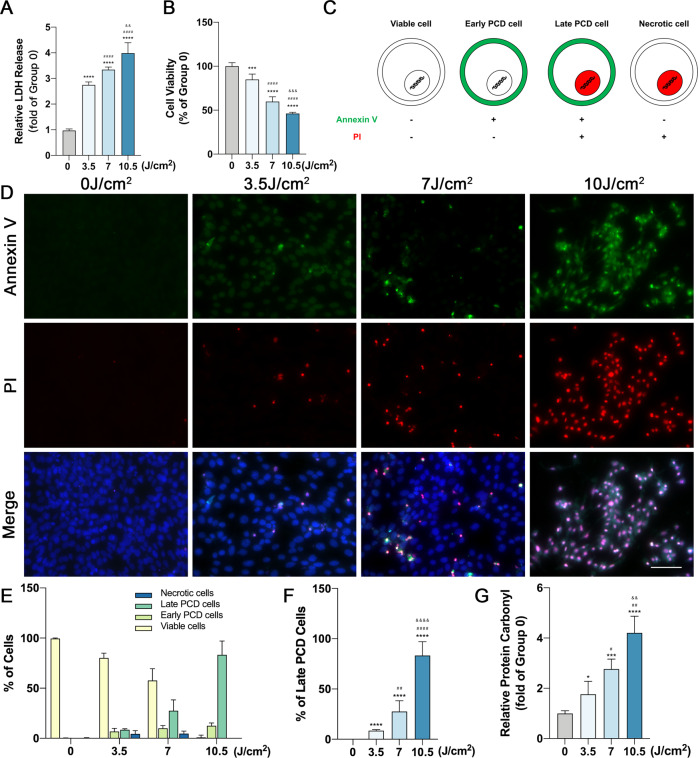
UVA induces 661w cells cell death and diminished cell viability. **A** LDH release analysis after UVA irradiation (*n* = 6 per group). **B** Cell viability analysis by MTT assay with different intensities (*n* = 6 per group). **C** Annexin V and PI co-staining pattern chart. **D** Representative images of annexin V (green) and PI (red) staining after UVA irradiation with the intensity of 0, 3.5, 7, 10.5 J/cm^2^ (Scale Bar: 20 μm) (*n* = 6 per group). **E** Analysis of cell death types after UVA irradiation (*n* = 6 per group). **F** Analysis of late PCD cells after UVA irradiation (*n* = 6 per group). **G** ROS production analysis by testing the amount of protein carbonyl with different intensities (*n* = 6 per group). Statistical significance was analyzed with the unpaired Student’s t-test. Statistical significance of compared with 0 J/cm^2^, 3.5 J/cm^2^, 7 J/cm^2^ is indicated as ^*^, ^#^ and ^&^. *P, ^#^*P* or ^&^*P* < 0.05, **P, ^##^*P* or ^&&^*P* < 0.01, ***P, ^###^*P* or ^&&&^*P* < 0.001, ****P, ^####^*P* or ^&&&&^*P* < 0.0001. All values are expressed as mean ± SD.

To investigate the role of UVA in photoreceptor cells, we set up an in vitro UVA injury model of a photoreceptor cell line (661 W) consisting of 4 different radiation intensities (0, 3.5, 7, and 10.5 J/cm^2^). After UVA exposure for 24 h, we observed a superabundant ROS production (Fig. 1G). Meanwhile, the levels of lactate dehydrogenase (LDH) released were 2.7 to 4.0-fold higher than the control group, which indicated a continuous increase in cell death as UVA doses increased (Fig. 1A). Similarly, the MTT results demonstrated a decrease in cellular activity (Fig. 1B).

### RIPK3 is involved in UVA-induced photoreceptor cell death

**Fig. 2:**
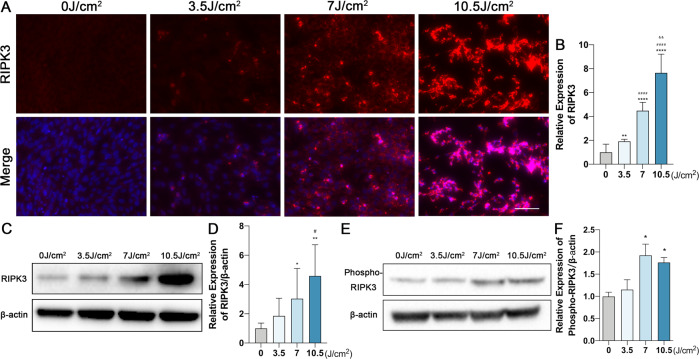
RIPK3 expression is upregulated by UVA in 661w cells. **A**, **B** Representative images and analysis of RIPK3 expression by immunofluorescence staining (Scale Bar: 20 μm) (*n* = 6 per group). **C**, **D** Representative images and analysis of RIPK3 expression by Western Blot (*n* = 6 per group). **E**, **F** Representative images and analysis of Phospho-RIPK3 expression by Western Blot (*n* = 3 per group). Statistical significance was analyzed with the unpaired Student’s t-test. Statistical significance of compared with 0 J/cm^2^, 3.5 J/cm^2^, 7 J/cm^2^ is indicated as ^*^, ^#^ and ^&^. *P, ^#^*P* or ^&^*P* < 0.05, **P, ^##^P or ^&&^P < 0.01, ***P, ^###^*P* or ^&&&^*P* < 0.001, ****P, ^####^*P* or ^&&&&^*P* < 0.0001. All values are expressed as mean ± SD.

**Fig. 3:**
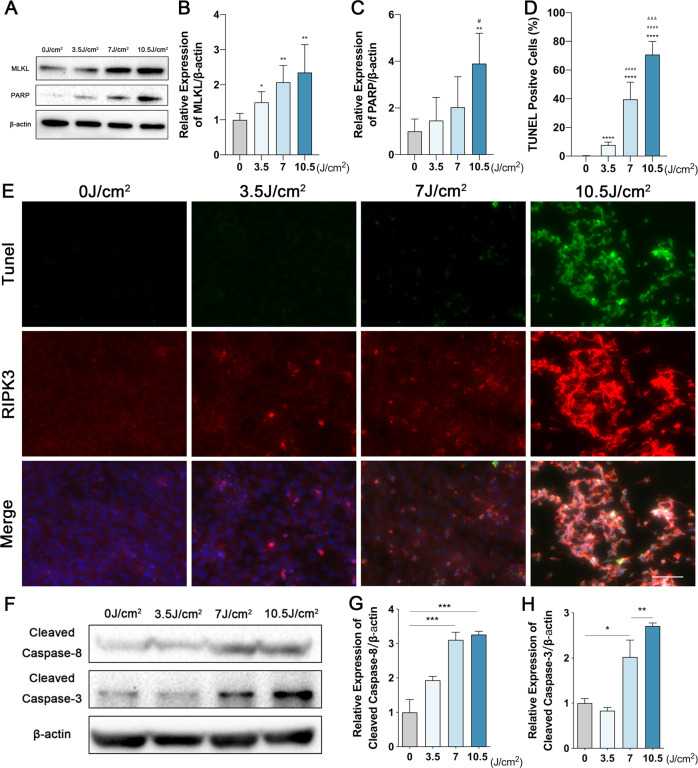
MLKL is involved in RIPK3-mediated necroptosis after UVA irradiation. **A**–**C** Representative images and analysis of MLKL and PARP expression by Western Blot (*n* = 4–5 per group). **D**, **E** Representative images and analysis of TUNEL (green) and RIPK3 (red) expression by co-staining (Scale Bar: 20 μm) (*n* = 6 per group). **F**–**H** Representative images and analysis of Cleaved Caspase-8 and Cleaved Caspase-3 expression by Western Blot (*n* = 3 per group). Statistical significance was analyzed with the unpaired Student’s t-test. Statistical significance of compared with 0 J/cm^2^, 3.5 J/cm^2^, 7 J/cm^2^ is indicated as ^*^, ^#^ and ^&^. *P, ^#^*P* or ^&^*P* < 0.05, **P, ^##^*P* or ^&&^*P* < 0.01, ***P, ^###^*P* or ^&&&^*P* < 0.001, ****P, ^####^P or ^&&&&^P < 0.0001. All values are expressed as mean ± SD.

To further clarify the mechanism of UVA-induced photoreceptor injury, we examined the stage of cell death by annexin V and propidium iodide (PI) staining. Annexin V positive and PI negative staining was traditionally used to identify early apoptotic cell death, but early stages of necroptosis were shown to also display the same staining pattern [46, 47]. Therefore, we used the Annexin V/ PI to discriminate early and late PCD in our UVA-induced photoreceptor injury model. Interestingly, photoreceptors mostly exhibited a late PCD morphology [annexin V ( + ), PI ( + )], rather than early PCD [annexin V ( + ), PI (−)], necrotic [annexin V (−), PI ( + )] or viable morphology [annexin V (−), PI (−)] (Fig. 1C–F). This could represent both late apoptosis or late necroptosis. Moreover, RIPK3 expression was significantly up-regulated by Western blotting and Immunofluorescence (Fig. 2A–D), and UVA also induced a dose-dependent phosphorylation of RIPK3 (Fig. E, F). In contrast to RIPK3, we could not detect any variation in either RIPK1 or phosphorylated RIPK1 (ρ-RIPK1), which is the upstream activator of RIPK3 (Fig. S1A, B). However, MLKL and Poly (ADP-ribose) polymerase-1 (PARP), the key downstream partner of RIPK3 and the PCD marker, were significantly upregulated (Fig. 3A–C). The TUNEL/RIPK3 double-labeled staining further supported the evidence for necroptotic cell death of photoreceptors cells after UVA irradiation (Fig. 3D, E). Caspase 8 and Caspase 3 cleavage was also observed in a dose dependent manner, indicating that apoptosis is also involved (Fig. 3F–H).

### RIPK3 inhibition suppresses UVA-induced cell death and necroptosis

**Fig. 4:**
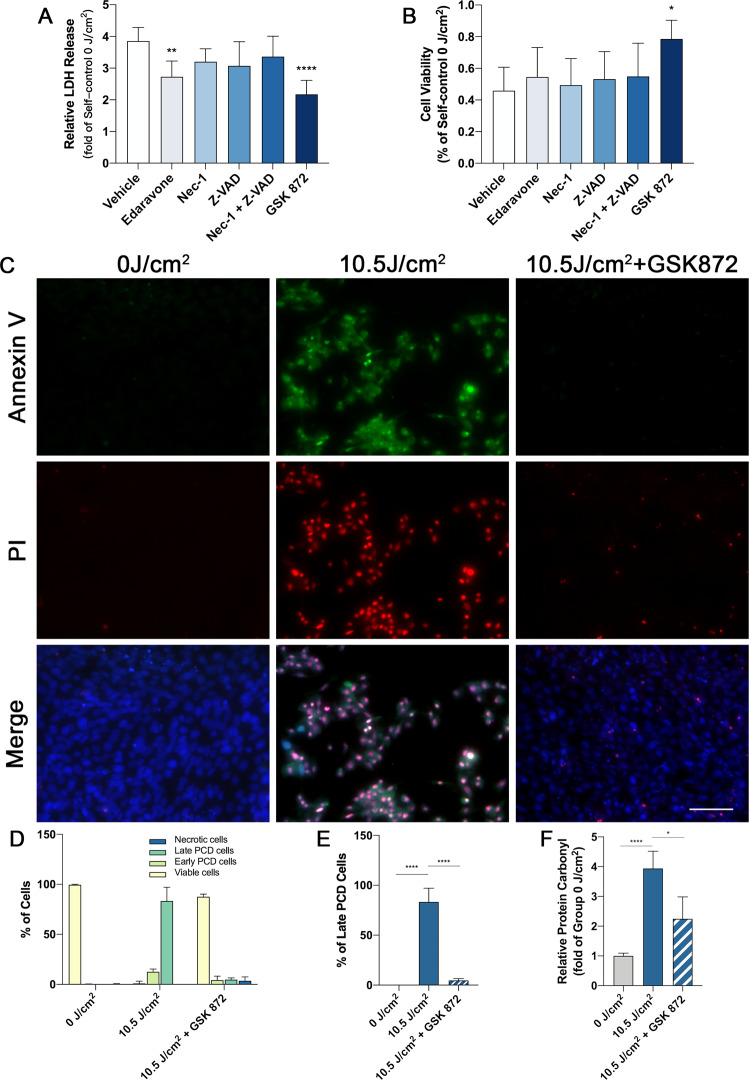
The inhibition of RIPK3 suppresses cell death and improved cell viability after high-dose UVA irradiation. **A** LDH release analysis of 661w cells treated with Endaravone (100 μM), Nec-1 (15 μM), Z-VAD (15 μM), combination of Nec-1 (15 μM) and Z-VAD (15 μM), GSK 872 (2 μM) after 10.5 J/cm^2^ UVA irradiation (*n* = 6 per group). **B** Cell viability analysis of 661w cells treated with Endaravone (100 μM), Nec-1 (15 μM), Z-VAD (15 μM), combination of Nec-1 (15 μM) and Z-VAD (15 μM), GSK 872 (2 μM) after 10.5 J/cm^2^ UVA irradiation by MTT assay (*n* = 6 per group). **C** Representative images of annexin V (green) and PI (red) staining in cells treated with GSK 872 (Scale Bar: 20 μm) (*n* = 6 per group). **D** Analysis of cell death types in cells treated with GSK 872 (*n* = 6 per group). **E** Analysis of late PCD cells in cells treated with GSK 872 (*n* = 6 per group). **F** ROS production analysis by testing the amount of protein carbonyl in cells treated with GSK 872 after 10.5 J/cm^2^ UVA irradiation (*n* = 6 per group). Statistical significance was analyzed with the unpaired Student’s t-test. Statistical significance of compared with 0 J/cm^2^, 3.5 J/cm^2^, 7 J/cm^2^ is indicated as ^*^, ^#^ and ^&^. *P, ^#^*P* or ^&^*P* < 0.05, **P, ^##^*P* or ^&&^*P* < 0.01, ^*^**P, ^###^*P* or ^&&&^P < 0.001, ****P, ^####^*P* or ^&&&&^*P* < 0.0001. All values are expressed as mean ± SD.

**Fig. 5:**
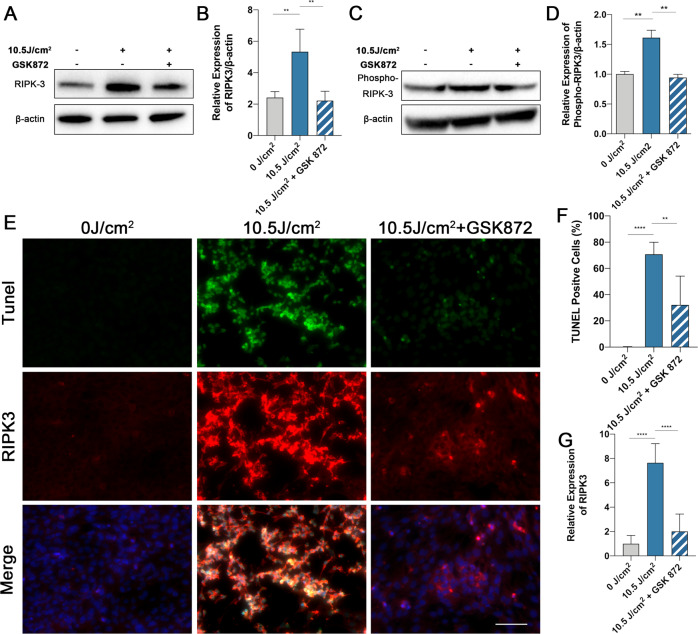
The level of cell death is attenuated under the inhibition of RIPK3 protein expression. **A**, **B** Representative images and analysis of RIPK3 expression by Western Blot in cells treated with GSK 872 after 10.5 J/cm^2^ UVA irradiation (*n* = 4 per group). **C**, **D** Representative images and analysis of Phospho-RIPK3 expression by Western Blot in cells treated with GSK 872 after 10.5 J/cm^2^ UVA irradiation (*n* = 3 per group). **E**–**G** Representative images and analysis of TUNEL (green) and RIPK3 (red) expression by co-staining in cells treated with GSK 872 after 10.5 J/cm^2^ UVA irradiation (Scale Bar: 20 μm) (*n* = 6 per group). Statistical significance was analyzed with the unpaired Student’s t-test. Statistical significance is indicated as ^*^. *P < 0.05, **P < 0.01, ****P < 0.0001. All values are expressed as mean ± SD.

**Fig. 6:**
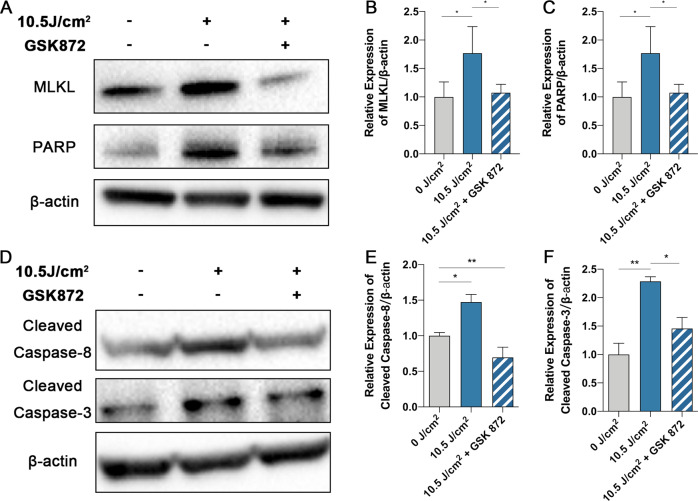
The level of necroptosis is attenuated under the inhibition of RIPK3 protein expression. **A**–**C** Representative images and analysis of MLKL, PARP expression by Western Blot in cells treated with GSK 872 after 10.5 J/cm^2^ UVA irradiation (*n* = 4 per group). **D**–**F** Representative images and analysis of Cleaved Caspase-8 and Cleaved Caspase-3 expression by Western Blot in cells treated with GSK 872 after 10.5 J/cm^2^ UVA irradiation (*n* = 4 per group). Statistical significance was analyzed with the unpaired Student’s t-test. Statistical significance was analyzed with the unpaired Student’s t-test. Statistical significance is indicated as ^*^. *P < 0.05, **P < 0.01. All values are expressed as mean ± SD.

To address the effect of RIPK3 inhibition on UVA-induced necroptosis, we used various small molecule inhibitors of different components of the cell death pathways (RIPK3–GSK 872, ROS-Edaravone, RIPK1–Nec-1, CASPASE–Z-VAD-FMK,) to assess cell viability after high-dose UVA exposure. Free radical scavenger Edaravone and RIPK3 inhibitor GSK872 but not RIPK1 or caspase inhibitors suppressed LDH release. In MTT assay only GSK872 was able to reach statistical significance whereas Edaravone showed a trend for protection but did not reach statistical significance. (Fig. 4A, B). Treatment with RIPK3 inhibitor GSK 872 effectively reduced the amount of late-stage cell death by annexin and PI co-staining (Fig. 4C–E). Additionally, ROS production was significantly reduced with GSK 872 treatment (Fig. 4F). Western blotting and Immunofluorescence also showed downregulation of RIPK3, lower RIPK3 phosphorylation and less TUNEL positive cells (Fig. 5A–G). Compared to UVA irradiation alone, RIPK3 inhibition decreased the expression of necroptosis-related proteins MLKL and PARP (Fig. 6A–C). Interestingly, RIPK3 inhibition with GSK872 reduced the levels of Caspase 8 and Caspase 3 cleavage, suggesting that RIPK3 might also modulate apoptotic cell death in this model (Fig. 6D–F).

### RIPK3 deficient mouse retina is resistant to UVA-induced damage

**Fig. 7:**
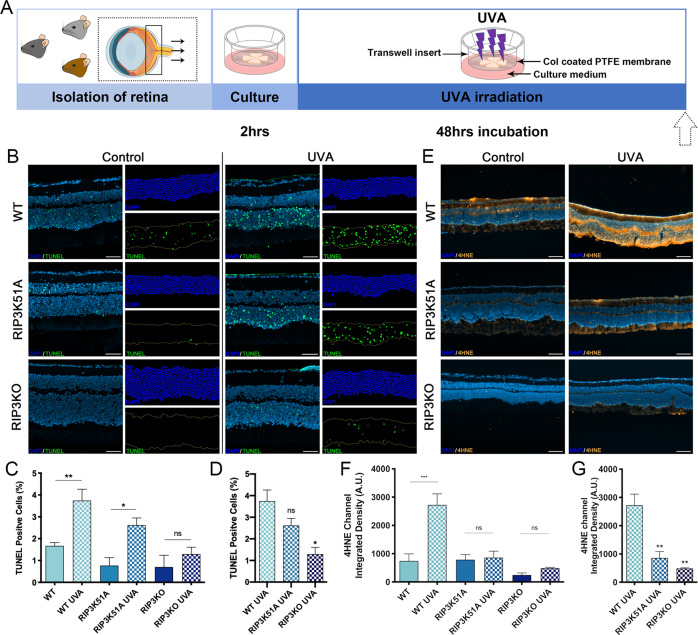
Knockout of RIPK3 reduces the number of cell death and the level of oxidative stress. **A** Flow chart of retina explant culture ex vivo and UVA irradiation model. **B**–**D** Representative images and analysis of sections stained with DAPI (Blue) and TUNEL (Green) from WT, RIP3K51A and RIP3KO mouse retinal explants, showing control and treatment (UVA 10.5 J/cm2) groups (*n* = 3). Scale bar = 50 µm. Image J post processed images are displayed next to each original picture. **E**–**G** Representative images and analysis of sections stained with DAPI (Blue) and 4HNE (Orange) from WT, RIP3K51A and RIP3KO mouse retinal explants, showing control and treatment (UVA 10.5 J/cm^2^) groups (*n* = 3). Scale bar = 100 µm. Statistical significance was analyzed with ANOVA followed by Bonferroni’s multiple comparison test between selected groups. ns = not significant; * = *p* < 0.05; ** = *p* < 0.01; ***=*p* < 0.001.

To verify that our results are not limited to this specific photoreceptor cell line, we used a model of retinal explants culture to assess the effects of UVA irradiation on mouse photoreceptors (Fig. 7A). Oxidative stress was measured by the reactive aldehyde 4-hydroxynoneal (4HNE) immunostaining, a major biomarker of lipid peroxidation [48, 49]. UVA irradiation caused increased retinal oxidative stress in wild type (WT) explants, but not in RIP3 kinase dead (RIPK3K51A) or RIPK3 knockout (RIPK3^−/−^) explants (Fig. 7E–G). We then proceeded with the TUNEL staining, and UVA caused a marked increase in WT photoreceptor cell death, while RIPK3K51A showed a trend to protect compared to WT, but without significant difference, and RIP3K^−/−^ rescued almost completely the explants from cell death in these assay conditions (Fig. 7B–D).

## Discussion

UVA is the most penetrating ultraviolet light, which can reach the retina directly through the anterior part [8]. Although several studies have reported the effects of UV radiation on the retina, the mechanisms of UVA damage to photoreceptors have not been clearly elucidated. Here, we focused on the pathogenesis of UVA induced damage/injury on photoreceptors and identified a significant role of RIPK3, but not RIPK1, on PCD. The connection between UV irradiation and RIPK3 upregulation has not been reported before for any retinal cell type, and establishes a novel connection between UV irradiation and photoreceptor cell injury.

In our results, we detected that UVA induces photoreceptor cell death in the form of necroptosis along with the upregulation of RIPK3 but not RIPK1. Generally, necroptosis is regulatory necrosis that heavily relies on RIPK1/3-mediated complexes containing MLKL. RIPK1/3 are commonly expressed and widely known for their role in promoting cell death, especially necroptosis [50, 51]. Interestingly, UVA irradiation only mediated the high expression of RIPK3 in photoreceptor cells. RIPK3 activation leads to MLKL oligomerization and phosphorylation, and eventually to the execution of necroptosis [41, 52, 53]. Notably, neither RIPK1 nor phosphorylated RIPK1 were involved in UVA-induced necroptosis. Simultaneously, our model shows that UVA also induces apoptosis, by the increased activation of Caspase-8 and Caspase-3. Activated Caspase-8 mainly delivers the death signals to executor caspases including Caspase-3, which then cleave various cellular proteins to complete the process [54, 55].

In the present study, UVA irradiation was associated with upregulation of PARP, a nuclear enzyme essential for DNA repair [56]. PARP plays multiple cellular roles and can exert a protective function against UV irradiation-induced injury [57–59]. Furthermore, our study confirmed and furthered strengthened the observation that UVA radiation induces oxidative stress and ultimately cell death through the accumulation of ROS which play a pivotal role in the necropolis pathway [60, 61].

Next, we tried to suppress UVA damage by inhibiting multiple injurious pathways. We applied ROS scavenger Edaravone, RIPK1 inhibitor Nec-1, Pan-caspase inhibitor Z-VAD and RIPK3 inhibitor GSK 872. Edaravone is a free radical scavenger, used clinically in Japan, which exerts an early neuroprotective effect through antioxidative and anti-inflammatory pathways [62]. Nec-1 is a small-molecule inhibitor generally applied to disease models to examine RIPK1 contribution in cell death and inflammation, while Z-VAD is a pan-CASPASE (apoptosis) inhibitor [63–65]. GSK 872 is a cell-permeable quinolinyl-benzothiazolamine compound that is reported to act as a RIPK3-selective kinase inhibitor, with a selectivity of more than 1000 times higher than its affinity for 300 other kinases, including RIPK1 [66, 67]. Our results showed that GSK 872 administration significantly dampened UVA-induced cell death and viability reduction by inhibiting necroptosis levels. To further explore the action of GSK872, we correlated RIPK3 downregulation with a decrease in TUNEL positive cells after GSK872 administration. These findings indicate that necroptosis was suppressed by RIPK3 inhibition. Expression of MLKL, the downstream of RIPK3 mediator, was reduced as well. Besides that, PARP expression and ROS production were at a lower level. These results suggested that RIPK3 responds in a major role to UVA radiation and participates in the regulation of oxidative stress and DNA fragmentation. Taken together, the photoreceptor cells irradiated under UVA were rescued when RIPK3 activity was inhibited by GSK 872. Our findings were further confirmed in an ex-vivo retinal explant model, showing that genetic ablations of RIPK3 significantly reduced UVA-induced damage. Interestingly, kinase dead RIPK3 mice (_RIPK3_^K51A^) did not show as much protection from UVA-induced cell death as the complete absence of RIPK3, suggesting that the role of RIPK3 in modulating cell death in this model may not be limited to its kinase function. This observation is in accordance to previously published data showing that in certain scenarios kinase dead RIPK3 could specifically induce apoptosis by recruiting a RIPK1-FADD-caspase 8 platform and activating caspase 8 and caspase 3 [68].

**Fig. 8:**
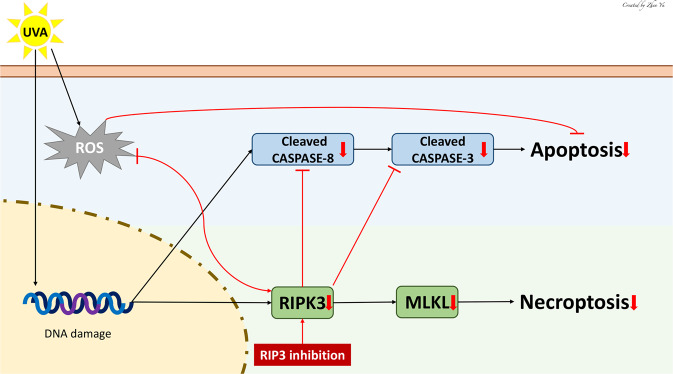
Schematic summary for the present study. UVA induced ROS production, DNA damage, along with RIPK3 activation and up regulation. RIPK3 but not RIPK1 in turn induces necroptosis by upregulation of MLKL expression and suppresses apoptosis by downregulation of Cleaved Caspase-8 and Cleaved Caspase-3. GSK 872 (RIPK3 inhibitor) can significantly attenuate UVA-induced cell death via RIPK3-mediated pathway.

To our knowledge, this is the first study to reveal the in vitro and ex-vivo effects of RIPK3 in UVA-induced photoreceptor cell death and necroptosis. Interestingly, RIPK1 did not appear to contribute to cell death and necroptosis after UVA irradiation. Neither Z-VAD alone nor in combination with Nec-1 (Z-VAD + Nec-1) significantly reduced the level of cell death, whereas RIPK3 blockade alone or its absence did reduce cell death. This supports the hypothesis that UVA induces necroptosis through RIPK3, bypassing RIPK1. Thus, RIPK3 may comprise a potential target for UVA-induced cell injury. Figure 8 summarizes our results and shows a proposed mechanism of UVA-induced photoreceptor cell death. Although these findings are yet to be verified in vivo, for the first time this study highlight the central role of RIPK3 in photoreceptor cell death via MLKL-mediated necroptosis and ROS-mediated apoptosis in UVA-induced photoreceptor cell death.

## Materials and methods

### Animals

All animal procedures complied with the Association for Research in Vision and Ophthalmology (ARVO) Statement for the Use of Animals in Ophthalmic and Vision Research under the approval by the Animal Care Committee of Massachusetts Eye and Ear Infirmary. RIPK3^K51A^ mice were kindly provided by GlaxoSmithKline. RIPK3^−/−^ mice and wild-type C57BL/6 J mice were purchased from the Jackson Laboratory. For the experiments, animals between 8 and 12 weeks old were used. All the mice were fed chow and water on standard laboratory diet with a 12 h light/12 h dark cycle in an air-controlled room.

### Cell culture

The photoreceptor cell line 661 W was friendly provided by Dr Miayyad Al-Ubaidi (University of Houston, TX, USA) [69]. Cells were cultured in a 37 °C humidified incubator under 5% CO_2_ and 95% atmosphere. The 661 W cells were maintained in Dulbecco’s modified Eagle’s medium (DMEM) containing 10% fetal bovine serum (FBS), 100 U/mL penicillin, 100 U/mL streptomycin, a final concentration of 1 mM Sodium Pyruvate, 0.1 mM hydrocortisone sodium succinate. Cells were passaged by trypsinization with 0.25% EDTA trypsin every 2–4 day and cultured in 6-well plates with 2 mL medium or in 96-well plates with 150 μL medium.

### Treatment and UVA light irradiation model

The 661w cells were seeded at a concentration of 2.0 × 10^5^ cells/mL in a 6-well plate and cultured for 48 h until confluency. RIPK3 inhibitor GSK 872 (2 μM final concentration) (Sigma-Aldrich, MO, USA) diluted with DMSO (Sigma-Aldrich) and DMSO vehicles (0.01% final DMSO concentration) were performed in the culture medium 1 h before UVA irradiation. The plates were placed in a chamber with constant temperature of 37 °C and irradiated with UVA tubes (365 nm wavelength, XX- 15BLB; Analytik Jena, MA, USA) at a distance of 13 cm. The total irradiation energy was 0 J/cm^2^ (0 minute 0 s), 3.5 J/cm^2^ (15 min), 7.0 J/cm^2^ (30 min) and 10.5 J/cm^2^ (45 min) with a radiation intensity of 3.89 mW/cm2, which was measured by an UVA sensor (UVA/B Light Meter 850009, Sper Scientific AZ, USA). The cells in culture medium were harvested 24 h after irradiation. Retinal explants were irradiated with 10.5 J/cm^2^ and incubated for 48 h after irradiation.

### LDH cytotoxicity assay

LDH release assay was performed following the manufacturer’s protocol (LDH Cytotoxicity Assay Kit; Cell Biolabs, CA, USA). Briefly, at 24 h post UVA irradiation, by treating with 15 μL triton X-100 solution for 10 min, these wells were set as a positive control to release all the LDH in the cells. A total of 90 μL of cell culture medium and 10 μL LDH substrate were transferred and mixed into a 96-well plate. The mixture was incubated at 37 °C and 5% CO_2_ in the dark until the OD 450 values of positive control wells no longer increased. The 96-well plate was read at 450 nm wavelength using an absorbance microplate reader (Molecular Devices, CA, USA). The levels of LDH release were standardized with the positive control as 100% cell death.

### Cell viability assay

MTT cell proliferation assay kit (MTT Cell Proliferation Assay; Cell Biolabs) was used for testing 661w cells viability on the basis of the manufacturer’s protocol. In short, 10 μL of the CytoSelectTM MTT Cell Proliferation Assay Reagent was added to each well after 24 h post UVA irradiation. Then, the plate was incubated at 37 °C and 5% CO_2_ for 2 h until purple precipitate was visible under microscope. After adding 100 μL of Detergent Solution into each well, the plate was incubated for 2 h protected from light. The plate was read at 545 nm wavelength using an absorbance microplate reader (Molecular Devices).

### Protein carbonyl ELISA

The amount of protein carbonyl in the cells samples was measured by a protein carbonyl ELISA kit (OxiSelect; Cell Biolabs) and performed following the manufacturer’s protocol. The plate was read at 450 nm wavelength using an absorbance microplate reader (Molecular Devices).

### Annexin V and PI staining

661w cells were seeded in a sterile 8-well glass slide (Sigma-Aldrich) and irradiated by UVA based on above description. 24 hours later, slides were washed twice with cold cell staining buffer (Apoptosis Detection Kit, BioLegend, CA, USA) and added 100 μL culture medium with 5 μL of FITC annexin V and 10 μL of Propidium Iodide Solution. The slides were gently rotated and incubated for 15 min at room temperature and protected from light. Images were captured using a microscope Axio imager M2 (M2, Zeiss, Oberkochen, Germany).

### 661 W Cells Immunofluorescence and TUNEL staining

The irradiated cells were cultured in a sterile 8-well glass slide (Sigma-Aldrich) and irradiated by UVA based on above description. Cell slides were fixed in 4% PFA for 15 min, blocked with 1% bovine serum albumin (BSA) with 0.4% Triton X-100 in PBS for 1 h at room temperature (RT). The slides were incubated with primary antibodies (Table S1) overnight at 4 °C. Subsequently, the slides were washed with PBS for 5 min 3 times, incubated with secondary antibodies (Thermo Fisher Scientific, Waltham, MA, USA) for 1 h at RT, washed 3 times with PBS, and coverslipped using PloLong Gold Antifade Reagent (Thermo Fisher Scientific). For TUNEL staining, the assay was performed by using the ApopTag Plus In Situ Apoptosis Fluorescein Detection Kit (Thermo Fisher Scientific), which utilizes a fluorescein-conjugated anti-digoxigenin antibody. Images were captured using a microscope Axio imager M2 (M2, Zeiss, Oberkochen, Germany).

### Western blot

After UVA irradiation, the cells were respectively homogenized in pre-chilled mammalian protein extraction reagent (M-PER, Thermo Fisher Scientific) with protease/phosphatase inhibitor cocktail (cOmplete Mini, IN, USA). Samples concentrations were measured by Coomassie (Bradford) Protein Assay Kit (Thermo Fisher Scientific). The samples were heated in NuPAGE Sample Buffer (Thermo Fisher Scientific) containing 5% Tris(2-carboxyethyl) phosphine hydrochloride solution (Sigma-Aldrich) at 95 °C for 5 min and electrophoresed using NuPAGE Bis-Tris Gels (Thermo Fisher Scientific) at 120 V. Next, the transferred polyvinylidene difluoride membranes (Sigma-Aldrich) were blocked and incubated with primary antibodies at 4 °C overnight followed by labeling with HRP-conjugated secondary antibodies at room temperature for 1 h. The incubated membranes were developed with HRP substrate reagent (Genetex, CA, USA) and recorded with an ECL imaging system ChemiDoc MP (Bio-Rad Laboratories, CA, USA).

### Retinal explant culture

Mice were euthanized by CO_2_ inhalation followed by cervical dislocation and eyeballs were immediately enucleated using westcott scissors under dissecting microscope removing most of the extraocular tissues. Eyeballs were submerged in 70% ethanol for 10 s and then placed in PBS. Under cell culture hood with a dissecting microscope each eyeball was placed in a 35 mm culture dish containing 2 ml of DMEM (Thermo Fisher 12430-054) containing 1 mM Sodium Pyruvate and 20% heat inactivated FBS (retina medium). Eyeballs were pierced with a number 11 scalpel at the limbus and a curved micro scissors was used to separate the anterior segment. The lens was removed, and four radial cuts were made on the posterior segment cutting all the way through the sclera. The petaloid posterior segment was then incubated in a new 35 mm dish with fresh and warm retina medium in a cell incubator for 1 h, to make the separation between retina and RPE less traumatic. After incubation the retina is easily peeled from the RPE, but it is important to not touch the retina during any point of the tissue manipulation. The retinal attachment to the optic nerve was cut using a curved micro scissors with care not to touch the surrounding tissue. After retinal separation, the retina was aspirated and transferred with a sterile transfer pipette to a 12 mm insert collagen coated 0.4 µm PTFE membrane Transwell within a 12 well plate (COSTAR #3493, Corning, ME, USA) with the photoreceptors facing the membrane. The petals were open carefully with a sterile blunt instrument, the remaining retinal medium inside the inner Transwell chamber was aspirated and 600 microliter of fresh warm retinal medium was added to the outer well. The retinal explants were then incubated for 1 h at 37 °C 5%CO_2_ before UVA treatment 10.5 J/cm^2^. After UVA treatment retinal explants were incubated again, had the media exchanged after 24 h, and incubated for another 24 h for a total of 48 h after UVA treatment. The right eye of each animal was used for the treatment, and the left eye for the control. Control explants were also incubated for 48 h and had the media exchanged at 24 h.

### Retinal explant slides preparation

Retinal explants were fixed while attached to the transwell membrane in freshly prepared 4% paraformaldehyde for 30 minutes, then washed 3 times with PBS for 5 min. Cryoprotection was conducted with 15%, 20%, and 30% sucrose in PBS for 1 h each. Sucrose solution was removed and Optimal Cutting Temperature (OCT) Compound (Tissue-Plus, Fisher HealthCare, TX, USA) was added on top of explants inside the transwell. The whole transwell was then submerged in a 2-methylbutane (Sigma) Becker inside an insulated foam container with liquid nitrogen until OCT was fully solidified. Transwell membrane was separated from the plastic support with a number 11 scalpel, and the OCT block containing the retinal explant was then removed for cryosectioning using Leica CM1860 Cryostat. Sections of 10–12 μm were transferred to Colorfrost Plus microscope slides (Thermo Scientific) and stored at −20 °C.

### Retinal Explant 4HNE Immunofluorescence

Slides were washed two times with PBS to remove the OCT compound, then sections were permeabilized with Triton X-100 0.1% for 10 min and rinsed again with PBS two more times. Slides were blocked in 5% BSA in PBS at room temperature for 1 h and incubated with 4HNE antibody 1:100 overnight at 4 °C. Slides were then washed 3 times with PBS for 5 min, then incubated with Alexa fluor 555 conjugated secondary antibody 1:200 for 1 h at 37 °C. Slides were posteriorly washed 3 times with PBS and coverslipped with VECTASHIELD Antifade mounting media with DAPI (Vector Laboratories, CA, USA). Images were captured using a microscope Axio imager M2 (M2, Zeiss, Oberkochen, Germany) using the same acquisition parameters, 1 image was acquired per section using a 10×/0.3 objective. The integrated density of each image was obtained after transforming images to 8-bit with ImageJ. Three sections were obtained and averaged per experiment per group, with a total of 3 experiments.

### Retinal explant TUNEL staining

The assay was performed using the ApopTag Plus In Situ Apoptosis Fluorescein Detection Kit (Thermo Fisher Scientific), which utilizes a fluorescein-conjugated anti-digoxigenin antibody, according to manufacturer’s protocol. Images were captured with Zeiss Axio Imager Z2 LSM 800 confocal microscope with 20×/0.8 objective, using the same acquisition parameter for all sections. Images were analyzed with ImageJ using an automatic macro script to count specifically the nuclei on the ONL on DAPI channel and then proceeding to count TUNEL ( + ) particles on FITC channel within the ONL to obtain the TUNEL ( + ) photoreceptor percentage. The same macro was applied to all images to avoid manual counting bias. Six sections were obtained and averaged per experiment per group, with a total of 3 experiments.

### Statistical analysis

Statistical analyses were performed by Prism software (GraphPad 8.0, CA, USA). Results were described as mean ± SD. Unpaired Student t-test was used to analyze statistical differences between two groups. ANOVA followed with post-hoc Tukey HSD test was used to multiple groups. The significance differences were defined as *P* < 0.05 (*), *P* < 0.01 (**), *P* < 0.001 (***) and *P* < 0.0001 (****).

## Supplementary information

Supplementary Legend

Authors list acknowldgement

Original western blots

Figure S1

Table S1

## Data Availability

The datasets used and/or analyzed during the current study are available from the corresponding author on reasonable request.

## References

[1] Bais AF, McKenzie RL, Bernhard G, Aucamp PJ, Ilyas M, Madronich S (2015). Ozone depletion and climate change: impacts on UV radiation. Photochem Photobio Sci.

[2] Sliney DH (2001). Photoprotection of the eye - UV radiation and sunglasses. J Photochem Photobiol B.

[3] Yam JC, Kwok AK (2014). Ultraviolet light and ocular diseases. Int Ophthalmol.

[4] Roduit R, Schorderet DF (2008). MAP kinase pathways in UV-induced apoptosis of retinal pigment epithelium ARPE19 cells. Apoptosis.

[5] Patton WP, Chakravarthy U, Davies RJ, Archer DB (1999). Comet assay of UV-induced DNA damage in retinal pigment epithelial cells. Invest Ophthalmol Vis Sci.

[6] Kaarniranta K, Pawlowska E, Szczepanska J, Jablkowska A, Blasiak J (2019). Role of mitochondrial DNA damage in ROS-mediated pathogenesis of age-related macular degeneration (AMD). Int J Mol Sci.

[7] Liang FQ, Godley BF (2003). Oxidative stress-induced mitochondrial DNA damage in human retinal pigment epithelial cells: a possible mechanism for RPE aging and age-related macular degeneration. Exp Eye Res.

[8] Ivanov IV, Mappes T, Schaupp P, Lappe C, Wahl S (2018). Ultraviolet radiation oxidative stress affects eye health. J Biophotonics.

[9] Sinha RP, Hader DP (2002). UV-induced DNA damage and repair: a review. Photochem Photobio Sci.

[10] O’Donovan P, Perrett CM, Zhang X, Montaner B, Xu YZ, Harwood CA (2005). Azathioprine and UVA light generate mutagenic oxidative DNA damage. Science.

[11] Karran P, Brem R (2016). Protein oxidation, UVA and human DNA repair. DNA Repair (Amst).

[12] Gechev TS, Van Breusegem F, Stone JM, Denev I, Laloi C (2006). Reactive oxygen species as signals that modulate plant stress responses and programmed cell death. Bioessays.

[13] Ong Tone S, Kocaba V, Bohm M, Wylegala A, White TL, Jurkunas UV (2021). Fuchs endothelial corneal dystrophy: The vicious cycle of Fuchs pathogenesis. Prog Retin Eye Res.

[14] Kwon YH, Fingert JH, Kuehn MH, Alward WL (2009). Primary open-angle glaucoma. N. Engl J Med.

[15] Lim LS, Mitchell P, Seddon JM, Holz FG, Wong TY (2012). Age-related macular degeneration. Lancet.

[16] Murakami Y, Matsumoto H, Roh M, Giani A, Kataoka K, Morizane Y (2014). Programmed necrosis, not apoptosis, is a key mediator of cell loss and DAMP-mediated inflammation in dsRNA-induced retinal degeneration. Cell Death Differ.

[17] Murakami Y, Matsumoto H, Roh M, Suzuki J, Hisatomi T, Ikeda Y (2012). Receptor interacting protein kinase mediates necrotic cone but not rod cell death in a mouse model of inherited degeneration. Proc Natl Acad Sci USA.

[18] Ueta T, Ishihara K, Notomi S, Lee JJ, Maidana DE, Efstathiou NE (2019). RIP1 kinase mediates angiogenesis by modulating macrophages in experimental neovascularization. Proc Natl Acad Sci USA.

[19] Jiang N, Zhang X, Gu X, Li X, Shang L (2021). Progress in understanding the role of lncRNA in programmed cell death. Cell Death Disco.

[20] Lockshin RA, Zakeri Z (2001). Programmed cell death and apoptosis: origins of the theory. Nat Rev Mol Cell Biol.

[21] Liu Y, Schiff M, Czymmek K, Talloczy Z, Levine B, Dinesh-Kumar SP (2005). Autophagy regulates programmed cell death during the plant innate immune response. Cell.

[22] Christofferson DE, Yuan J (2010). Necroptosis as an alternative form of programmed cell death. Curr Opin Cell Biol.

[23] Kajarabille N, Latunde-Dada GO (2019). Programmed cell-death by ferroptosis: antioxidants as mitigators. Int J Mol Sci.

[24] Kesavardhana S, Malireddi RKS, Kanneganti TD (2020). Caspases in cell death, inflammation, and pyroptosis. Annu Rev Immunol.

[25] Galluzzi L, Kroemer G (2008). Necroptosis: a specialized pathway of programmed necrosis. Cell.

[26] Negroni A, Cucchiara S, Stronati L (2015). Apoptosis, necrosis, and necroptosis in the gut and intestinal homeostasis. Mediators Inflamm.

[27] Trichonas G, Murakami Y, Thanos A, Morizane Y, Kayama M, Debouck CM (2010). Receptor interacting protein kinases mediate retinal detachment-induced photoreceptor necrosis and compensate for inhibition of apoptosis. Proc Natl Acad Sci USA.

[28] Molnar T, Mazlo A, Tslaf V, Szollosi AG, Emri G, Koncz G (2019). Current translational potential and underlying molecular mechanisms of necroptosis. Cell Death Dis.

[29] Pasparakis M, Vandenabeele P (2015). Necroptosis and its role in inflammation. Nature.

[30] Kaczmarek A, Vandenabeele P, Krysko DV (2013). Necroptosis: the release of damage-associated molecular patterns and its physiological relevance. Immunity.

[31] Zhang DW, Shao J, Lin J, Zhang N, Lu BJ, Lin SC (2009). RIP3, an energy metabolism regulator that switches TNF-induced cell death from apoptosis to necrosis. Science.

[32] He S, Wang L, Miao L, Wang T, Du F, Zhao L (2009). Receptor interacting protein kinase-3 determines cellular necrotic response to TNF-alpha. Cell.

[33] Cho YS, Challa S, Moquin D, Genga R, Ray TD, Guildford M (2009). Phosphorylation-driven assembly of the RIP1-RIP3 complex regulates programmed necrosis and virus-induced inflammation. Cell.

[34] Yang Z, Wang Y, Zhang Y, He X, Zhong CQ, Ni H (2018). RIP3 targets pyruvate dehydrogenase complex to increase aerobic respiration in TNF-induced necroptosis. Nat Cell Biol.

[35] Green DR (2019). The coming decade of cell death research: five riddles. Cell.

[36] Koo GB, Morgan MJ, Lee DG, Kim WJ, Yoon JH, Koo JS (2015). Methylation-dependent loss of RIP3 expression in cancer represses programmed necrosis in response to chemotherapeutics. Cell Res.

[37] Wu XN, Yang ZH, Wang XK, Zhang Y, Wan H, Song Y (2014). Distinct roles of RIP1-RIP3 hetero- and RIP3-RIP3 homo-interaction in mediating necroptosis. Cell Death Differ.

[38] Dondelinger Y, Aguileta MA, Goossens V, Dubuisson C, Grootjans S, Dejardin E (2013). RIPK3 contributes to TNFR1-mediated RIPK1 kinase-dependent apoptosis in conditions of cIAP1/2 depletion or TAK1 kinase inhibition. Cell Death Differ.

[39] Yu Z, Efstathiou NE, Correa V, Chen X, Ishihara K, Iesato Y (2021). Receptor interacting protein 3 kinase, not 1 kinase, through MLKL-mediated necroptosis is involved in UVA-induced corneal endothelium cell death. Cell Death Disco.

[40] Al-Moujahed A, Tian B, Efstathiou NE, Konstantinou EK, Hoang M, Lin H (2019). Receptor interacting protein kinase 3 (RIP3) regulates iPSCs generation through modulating cell cycle progression genes. Stem Cell Res.

[41] Sun L, Wang H, Wang Z, He S, Chen S, Liao D (2012). Mixed lineage kinase domain-like protein mediates necrosis signaling downstream of RIP3 kinase. Cell.

[42] Orozco S, Oberst A (2017). RIPK3 in cell death and inflammation: the good, the bad, and the ugly. Immunol Rev.

[43] Kataoka K, Matsumoto H, Kaneko H, Notomi S, Takeuchi K, Sweigard JH (2015). Macrophage- and RIP3-dependent inflammasome activation exacerbates retinal detachment-induced photoreceptor cell death. Cell Death Dis.

[44] Viringipurampeer IA, Shan X, Gregory-Evans K, Zhang JP, Mohammadi Z, Gregory-Evans CY (2014). Rip3 knockdown rescues photoreceptor cell death in blind pde6c zebrafish. Cell Death Differ.

[45] Xu J, Mo J, Liu X, Marshall B, Atherton SS, Dong Z (2018). Depletion of the receptor-interacting protein kinase 3 (RIP3) decreases photoreceptor cell death during the early stages of ocular murine cytomegalovirus infection. Invest Ophthalmol Vis Sci.

[46] Sawai H, Domae N (2011). Discrimination between primary necrosis and apoptosis by necrostatin-1 in Annexin V-positive/propidium iodide-negative cells. Biochem Biophys Res Commun.

[47] Krysko O, Aaes TL, Kagan VE, D’Herde K, Bachert C, Leybaert L (2017). Necroptotic cell death in anti-cancer therapy. Immunol Rev.

[48] Zarkovic N (2003). 4-hydroxynonenal as a bioactive marker of pathophysiological processes. Mol Asp Med.

[49] Liou GY, Storz P (2015). Detecting reactive oxygen species by immunohistochemistry. Methods Mol Biol.

[50] Ito Y, Ofengeim D, Najafov A, Das S, Saberi S, Li Y (2016). RIPK1 mediates axonal degeneration by promoting inflammation and necroptosis in ALS. Science.

[51] Wang Z, Jiang H, Chen S, Du F, Wang X (2012). The mitochondrial phosphatase PGAM5 functions at the convergence point of multiple necrotic death pathways. Cell.

[52] Zhao J, Jitkaew S, Cai Z, Choksi S, Li Q, Luo J (2012). Mixed lineage kinase domain-like is a key receptor interacting protein 3 downstream component of TNF-induced necrosis. Proc Natl Acad Sci USA.

[53] Seifert L, Werba G, Tiwari S, Giao Ly NN, Alothman S, Alqunaibit D (2016). The necrosome promotes pancreatic oncogenesis via CXCL1 and Mincle-induced immune suppression. Nature.

[54] Thornberry NA, Lazebnik Y (1998). Caspases: enemies within. Science.

[55] Someda M, Kuroki S, Miyachi H, Tachibana M, Yonehara S (2020). Caspase-8, receptor-interacting protein kinase 1 (RIPK1), and RIPK3 regulate retinoic acid-induced cell differentiation and necroptosis. Cell Death Differ.

[56] Ghodgaonkar MM, Zacal N, Kassam S, Rainbow AJ, Shah GM (2008). Depletion of poly(ADP-ribose) polymerase-1 reduces host cell reactivation of a UV-damaged adenovirus-encoded reporter gene in human dermal fibroblasts. DNA Repair (Amst).

[57] Pines A, Mullenders LH, van Attikum H, Luijsterburg MS (2013). Touching base with PARPs: moonlighting in the repair of UV lesions and double-strand breaks. Trends Biochem Sci.

[58] Robu M, Shah RG, Petitclerc N, Brind’Amour J, Kandan-Kulangara F, Shah GM (2013). Role of poly(ADP-ribose) polymerase-1 in the removal of UV-induced DNA lesions by nucleotide excision repair. Proc Natl Acad Sci USA.

[59] Chiu LY, Wu NL, Hung CF, Bai P, Dai YS, Lin WW (2021). PARP-1 involves in UVB-induced inflammatory response in keratinocytes and skin injury via regulation of ROS-dependent EGFR transactivation and p38 signaling. FASEB J.

[60] Karthikeyan R, Kanimozhi G, Prasad NR, Agilan B, Ganesan M, Srithar G (2018). Alpha pinene modulates UVA-induced oxidative stress, DNA damage and apoptosis in human skin epidermal keratinocytes. Life Sci.

[61] Zhao B, Shah P, Qiang L, He TC, Budanov A, He YY (2017). Distinct role of Sesn2 in response to UVB-induced DNA damage and UVA-induced oxidative stress in melanocytes. Photochem Photobiol.

[62] Zhang N, Komine-Kobayashi M, Tanaka R, Liu M, Mizuno Y, Urabe T (2005). Edaravone reduces early accumulation of oxidative products and sequential inflammatory responses after transient focal ischemia in mice brain. Stroke.

[63] Takahashi N, Duprez L, Grootjans S, Cauwels A, Nerinckx W, DuHadaway JB (2012). Necrostatin-1 analogues: critical issues on the specificity, activity and in vivo use in experimental disease models. Cell Death Dis.

[64] Shacham-Silverberg V, Sar Shalom H, Goldner R, Golan-Vaishenker Y, Gurwicz N, Gokhman I (2018). Phosphatidylserine is a marker for axonal debris engulfment but its exposure can be decoupled from degeneration. Cell Death Dis.

[65] Bagnjuk K, Stockl JB, Frohlich T, Arnold GJ, Behr R, Berg U (2019). Necroptosis in primate luteolysis: a role for ceramide. Cell Death Disco.

[66] Buchrieser J, Oliva-Martin MJ, Moore MD, Long JCD, Cowley SA, Perez-Simon JA (2018). RIPK1 is a critical modulator of both tonic and TLR-responsive inflammatory and cell death pathways in human macrophage differentiation. Cell Death Dis.

[67] Kaiser WJ, Sridharan H, Huang C, Mandal P, Upton JW, Gough PJ (2013). Toll-like receptor 3-mediated necrosis via TRIF, RIP3, and MLKL. J Biol Chem.

[68] Mandal P, Berger SB, Pillay S, Moriwaki K, Huang C, Guo H (2014). RIP3 induces apoptosis independent of pronecrotic kinase activity. Mol Cell.

[69] Tan E, Ding XQ, Saadi A, Agarwal N, Naash MI, Al-Ubaidi MR (2004). Expression of cone-photoreceptor-specific antigens in a cell line derived from retinal tumors in transgenic mice. Invest Ophthalmol Vis Sci.

