# Supplementary material for: UVA induces retinal photoreceptor cell death via receptor interacting protein 3 kinase mediated necroptosis
Source: Cell Death Discov. 2022 Dec 12;8:489. doi: 10.1038/s41420-022-01273-1 (PMC9744841; doi:10.1038/s41420-022-01273-1)
Supplement: Supplementary file 1 — Supplementary Legend [file 41420_2022_1273_MOESM1_ESM.docx]

**Supplementary legends**

**Fig. S1 RIPK1 is not involved in RIPK3-mediated necroptosis after UVA irradiation. A & B** Representative images and analysis of ρ-RIPK1 and RIPK1 expression by Western Blot (n=4 per group). *Statistical significance was analyzed with the unpaired Student's t-test. Statistical significance of compared with 0 J/cm^2^ is indicated as ns. ^ns^P ＞ 0.05. All values are expressed as mean ± SD.*
