# Supplementary material for: UVA induces retinal photoreceptor cell death via receptor interacting protein 3 kinase mediated necroptosis
Source: Cell Death Discov. 2022 Dec 12;8:489. doi: 10.1038/s41420-022-01273-1 (PMC9744841; doi:10.1038/s41420-022-01273-1)
Supplement: Supplementary file 2 — Authors list acknowldgement [file 41420_2022_1273_MOESM2_ESM.pdf]

Re: RESPONSE NEEDED - UVA-induced photoreceptor necroptosis paper

Efstathiou, Nikolaos <Nikolaos_Efstathiou@meei.harvard.edu>
Tue 11/22/2022 12:11 PM
To: Correa, Victor <Victor_Correa@MEEI.HARVARD.EDU>

**"I acknowledge the updated author list and accept the changes made to the manuscript"**

**NIKOLAOS E. EFSTATHIOU**

22 Νοε 2022, 12:08, ο χρήστης «Correa, Victor <Victor_Correa@meei.harvard.edu>» έγραψε:

Dear Colleagues,

During the review process of our paper, we have made some changes to the authors list. The updated authors list is as follows:


Zhen Yu*[1,2], Victor S. M. C. Correa*[2], Nikolaos E. Efstathiou[2], Henar Albertos-Arranz[3], Xiaohong Chen[2], Kenji Ishihara[2], Yasuhiro Iesato[2], Toshio Narimatsu[2], Dimitrios Ntentakis[2] and Demetrios G. Vavvas[2]

1 Shenzhen Eye Hospital, Jinan University, Shenzhen Eye Institute, Shenzhen, 518040, China.

2 Retina Service, Ines and Fred Yeatts Retina Research Laboratory, Angiogenesis Laboratory, Department of Ophthalmology, Massachusetts Eye and Ear, Harvard Medical School, Boston, MA, 02114, USA.

3 Department of Physiology, Genetics and Microbiology, University of Alicante, 03690 Alicante, Spain.

*These authors contributed equally to this work


You can access the manuscript submission with the following link: https://mts-cddiscovery.nature.com/cgi-bin/main.plex?el=A2Tf1BTR7D3CDj2F6A9ftdNqr1cRQfxGeKro8lLxoNPgZ

Please reply to acknowledge the updated author list and accepting the changes made to the manuscript
For your convenience, you can just copy the statement: **"I acknowledge the updated author list and accept the changes made to the manuscript"**.
Also, **don't forget to sign the email with your full name.**

Please let me know if you have any questions

Thank you in advance for your expedited response,


Victor San Martin Carvalho Correa, MD
Research Fellow.
Ines and Fred Yeatts Retina Research Laboratory, Mass Eye and Ear.
325 Cambridge Street, 3rd floor, Boston MA

Re: RESPONSE NEEDED - UVA-induced photoreceptor necroptosis paper

Vavvas, Demetrios,MD, PhD <Demetrios_Vavvas@meei.harvard.edu>
Tue 11/22/2022 12:15 PM
To: Correa, Victor <Victor_Correa@MEEI.HARVARD.EDU>
Cc: Zhen Ethan Yu <dryuzhen@hotmail.com>;Efstathiou, Nikolaos <Nikolaos_Efstathiou@meei.harvard.edu>;Henar <henar.albertos@gmail.com>;Kenji Ishihara <ikenx2@kuhp.kyoto-u.ac.jp>;Narimatsu, Toshio <Toshio_Narimatsu@MEEI.HARVARD.EDU>;Ntentakis, Dimitrios <Dimitrios_Ntentakis@MEEI.HARVARD.EDU>;Vavvas, Demetrios,MD, PhD <Demetrios_Vavvas@meei.harvard.edu>;iesato@shinshu-u.ac.jp <iesato@shinshu-u.ac.jp>;chenxh327@sysu.edu.cn <chenxh327@sysu.edu.cn>

YEs agree

______________________________


**Demetrios G. Vavvas MD, PhD**
*Solman and Libe Friedman Professor of Ophthalmology*
Director Retina Service | Harvard Medical School
Co-Director Ocular Regenerative Medical Institute
Principal Investigator Ines and Fredrick Yeatts Retinal Research Laboratory
Mass. Eye and Ear Infirmary and Mass General Hospital
243 Charles St. Boston MA 02114
**P** 617-573-6874 **E:** vavvas@meei.harvard.edu


On Nov 22, 2022, at 12:08 PM, Correa, Victor <Victor_Correa@MEEI.HARVARD.EDU> wrote:

Dear Colleagues,

During the review process of our paper, we have made some changes to the authors list. The updated authors list is as follows:


Zhen Yu*[1,2], Victor S. M. C. Correa*[2], Nikolaos E. Efstathiou[2], Henar Albertos-Arranz[3], Xiaohong Chen[2], Kenji Ishihara[2], Yasuhiro Iesato[2], Toshio Narimatsu[2], Dimitrios Ntentakis[2] and Demetrios G. Vavvas[2]

1 Shenzhen Eye Hospital, Jinan University, Shenzhen Eye Institute, Shenzhen, 518040, China.
2 Retina Service, Ines and Fred Yeatts Retina Research Laboratory, Angiogenesis Laboratory, Department of Ophthalmology, Massachusetts Eye and Ear, Harvard Medical School, Boston, MA, 02114, USA.
3 Department of Physiology, Genetics and Microbiology, University of Alicante, 03690 Alicante, Spain.
*These authors contributed equally to this work


You can access the manuscript submission with the following link: https://mts-cddiscovery.nature.com/cgi-bin/main.plex?el=A2Tf1BTR7D3CDj2F6A9ftdNqr1cRQfxGeKro8lLxoNPgZ

Please reply to acknowledge the updated author list and accepting the changes made to the manuscript
For your convenience, you can just copy the statement: **"I acknowledge the updated author list and accept the changes made to the manuscript"**.
Also, **don't forget to sign the email with your full name.**

Please let me know if you have any questions

Thank you in advance for your expedited response,


Victor San Martin Carvalho Correa, MD
Research Fellow.
Ines and Fred Yeatts Retina Research Laboratory, Mass Eye and Ear.
325 Cambridge Street, 3rd floor, Boston MA

**Re: RESPONSE NEEDED - UVA-induced photoreceptor necroptosis paper**

Henar Albertos <henar.albertos@gmail.com>
Tue 11/22/2022 12:29 PM
To: Narimatsu, Toshio <Toshio_Narimatsu@MEEI.HARVARD.EDU>
Cc: Correa, Victor <Victor_Correa@MEEI.HARVARD.EDU>;Vavvas, Demetrios,MD, PhD <Demetrios_Vavvas@meei.harvard.edu>;Zhen Ethan Yu <dryuzhen@hotmail.com>;Efstathiou, Nikolaos <Nikolaos_Efstathiou@meei.harvard.edu>;Kenji Ishihara <ikenx2@kuhp.kyoto-u.ac.jp>;Ntentakis, Dimitrios <Dimitrios_Ntentakis@MEEI.HARVARD.EDU>;iesato@shinshu-u.ac.jp <iesato@shinshu-u.ac.jp>;chenxh327@sysu.edu.cn <chenxh327@sysu.edu.cn>

External Email - Use Caution

Hi to everyone,

I acknowledge the updated author list and accept the changes made to the manuscript.

Thank you,
Henar Albertos Arranz

El mar, 22 nov 2022 a las 18:26, Narimatsu, Toshio (<Toshio_Narimatsu@meei.harvard.edu>) escribió:

Hi all,

I acknowledge the updated author list and accept the changes made to the manuscript.
Thank you.

Toshio Narimatsu

---

**From:** Vavvas, Demetrios,MD, PhD <Demetrios_Vavvas@meei.harvard.edu>
**Sent:** Tuesday, November 22, 2022 12:15
**To:** Correa, Victor <Victor_Correa@MEEI.HARVARD.EDU>
**Cc:** Zhen Ethan Yu <dryuzhen@hotmail.com>; Efstathiou, Nikolaos <Nikolaos_Efstathiou@meei.harvard.edu>; Henar <henar.albertos@gmail.com>; Kenji Ishihara <ikenx2@kuhp.kyoto-u.ac.jp>; Narimatsu, Toshio <Toshio_Narimatsu@MEEI.HARVARD.EDU>; Ntentakis, Dimitrios <Dimitrios_Ntentakis@MEEI.HARVARD.EDU>; Vavvas, Demetrios,MD, PhD <Demetrios_Vavvas@meei.harvard.edu>; iesato@shinshu-u.ac.jp <iesato@shinshu-u.ac.jp>; chenxh327@sysu.edu.cn <chenxh327@sysu.edu.cn>
**Subject:** Re: RESPONSE NEEDED - UVA-induced photoreceptor necroptosis paper

YEs agree

______________________________

**Demetrios G. Vavvas MD, PhD**
*Solman and Libe Friedman Professor of Ophthalmology*
Director Retina Service | Harvard Medical School
Co-Director Ocular Regenerative Medical Institute
Principal Investigator Ines and Fredrick Yeatts Retinal Research Laboratory
Mass. Eye and Ear Infirmary and Mass General Hospital
243 Charles St. Boston MA 02114
**P** 617-573-6874 **E:** vavvas@meei.harvard.edu

On Nov 22, 2022, at 12:08 PM, Correa, Victor <Victor_Correa@MEEI.HARVARD.EDU> wrote:

Dear Colleagues,

During the review process of our paper, we have made some changes to the authors list. The updated authors list is as follows:

Zhen Yu*[1,2], Victor S. M. C. Correa*[2], Nikolaos E. Efstathiou[2], Henar Albertos-Arranz[3], Xiaohong Chen[2], Kenji Ishihara[2], Yasuhiro Iesato[2], Toshio Narimatsu[2], Dimitrios Ntentakis[2] and Demetrios G. Vavvas[2]

1 Shenzhen Eye Hospital, Jinan University, Shenzhen Eye Institute, Shenzhen, 518040, China.
2 Retina Service, Ines and Fred Yeatts Retina Research Laboratory, Angiogenesis Laboratory, Department of Ophthalmology, Massachusetts Eye and Ear, Harvard Medical School, Boston, MA, 02114, USA.
3 Department of Physiology, Genetics and Microbiology, University of Alicante, 03690 Alicante, Spain.
*These authors contributed equally to this work

You can access the manuscript submission with the following link: https://mts-cddiscovery.nature.com/cgi-bin/main.plex?el=A2Tf1BTR7D3CDj2F6A9ftdNqr1cRQfxGeKro8lLxoNPgZ

Please reply to acknowledge the updated author list and accepting the changes made to the manuscript
For your convenience, you can just copy the statement: **"I acknowledge the updated author list and accept the changes made to the manuscript"**.
Also, **don't forget to sign the email with your full name.**

Please let me know if you have any questions

Thank you in advance for your expedited response,

Victor San Martin Carvalho Correa, MD
Research Fellow.
Ines and Fred Yeatts Retina Research Laboratory, Mass Eye and Ear.
325 Cambridge Street, 3rd floor, Boston MA

The information in this e-mail is intended only for the person to whom it is addressed.  If you believe this e-mail was sent to you in error and the e-mail contains patient information, please contact the Mass General Brigham Compliance HelpLine at https://www.massgeneralbrigham.org/complianceline .

--
**Henar Albertos-Arranz, Msc.**
Investigadora predoctoral FPU
*Neurobiología del Sistema Visual y terapia de enfermedades neurodegenerativas (NEUROVIS)*
Dpto. Fisiología, Genética y Microbiología
Universidad de Alicante
(Pabellón 13, ext. 1194; +34 630 306 072 1194)

Re: RESPONSE NEEDED - UVA-induced photoreceptor necroptosis paper

Narimatsu, Toshio <Toshio_Narimatsu@MEEI.HARVARD.EDU>
Tue 11/22/2022 12:26 PM
To: Correa, Victor <Victor_Correa@MEEI.HARVARD.EDU>
Cc: Vavvas, Demetrios,MD, PhD <Demetrios_Vavvas@meei.harvard.edu>;Zhen Ethan Yu <dryuzhen@hotmail.com>;Efstathiou, Nikolaos <Nikolaos_Efstathiou@meei.harvard.edu>;Henar <henar.albertos@gmail.com>;Kenji Ishihara <ikenx2@kuhp.kyoto-u.ac.jp>;Ntentakis, Dimitrios <Dimitrios_Ntentakis@MEEI.HARVARD.EDU>;iesato@shinshu-u.ac.jp <iesato@shinshu-u.ac.jp>;chenxh327@sysu.edu.cn <chenxh327@sysu.edu.cn>

Hi all,

I acknowledge the updated author list and accept the changes made to the manuscript.
Thank you.

Toshio Narimatsu

---

**From:** Vavvas, Demetrios,MD, PhD <Demetrios_Vavvas@meei.harvard.edu>
**Sent:** Tuesday, November 22, 2022 12:15
**To:** Correa, Victor <Victor_Correa@MEEI.HARVARD.EDU>
**Cc:** Zhen Ethan Yu <dryuzhen@hotmail.com>; Efstathiou, Nikolaos <Nikolaos_Efstathiou@meei.harvard.edu>; Henar <henar.albertos@gmail.com>; Kenji Ishihara <ikenx2@kuhp.kyoto-u.ac.jp>; Narimatsu, Toshio <Toshio_Narimatsu@MEEI.HARVARD.EDU>; Ntentakis, Dimitrios <Dimitrios_Ntentakis@MEEI.HARVARD.EDU>; Vavvas, Demetrios,MD, PhD <Demetrios_Vavvas@meei.harvard.edu>; iesato@shinshu-u.ac.jp <iesato@shinshu-u.ac.jp>; chenxh327@sysu.edu.cn <chenxh327@sysu.edu.cn>
**Subject:** Re: RESPONSE NEEDED - UVA-induced photoreceptor necroptosis paper

YEs agree

______________________________
**Demetrios G. Vavvas MD, PhD**
*Solman and Libe Friedman Professor of Ophthalmology*
Director Retina Service | Harvard Medical School
Co-Director Ocular Regenerative Medical Institute
Principal Investigator Ines and Fredrick Yeatts Retinal Research Laboratory
Mass. Eye and Ear Infirmary and Mass General Hospital
243 Charles St. Boston MA 02114
**P** 617-573-6874 **E:** vavvas@meei.harvard.edu

On Nov 22, 2022, at 12:08 PM, Correa, Victor <Victor_Correa@MEEI.HARVARD.EDU> wrote:

Dear Colleagues,

During the review process of our paper, we have made some changes to the authors list. The updated authors list is as follows:

Zhen Yu*[1,2], Victor S. M. C. Correa*[2], Nikolaos E. Efstathiou[2], Henar Albertos-Arranz[3], Xiaohong Chen[2], Kenji Ishihara[2], Yasuhiro Iesato[2], Toshio Narimatsu[2], Dimitrios Ntentakis[2] and Demetrios G. Vavvas[2]

1 Shenzhen Eye Hospital, Jinan University, Shenzhen Eye Institute, Shenzhen, 518040, China.
2 Retina Service, Ines and Fred Yeatts Retina Research Laboratory, Angiogenesis Laboratory, Department of Ophthalmology, Massachusetts Eye and Ear, Harvard Medical School, Boston, MA, 02114, USA.
3 Department of Physiology, Genetics and Microbiology, University of Alicante, 03690 Alicante, Spain.
*These authors contributed equally to this work

You can access the manuscript submission with the following link: https://mts-cddiscovery.nature.com/cgi-bin/main.plex?el=A2Tf1BTR7D3CDj2F6A9ftdNqr1cRQfxGeKro8lLxoNPgZ

Please reply to acknowledge the updated author list and accepting the changes made to the manuscript
For your convenience, you can just copy the statement: **"I acknowledge the updated author list and accept the changes made to the manuscript"**.
Also, **don't forget to sign the email with your full name.**

Please let me know if you have any questions

Thank you in advance for your expedited response,

Victor San Martin Carvalho Correa, MD
Research Fellow.
Ines and Fred Yeatts Retina Research Laboratory, Mass Eye and Ear.
325 Cambridge Street, 3rd floor, Boston MA

## Re: RESPONSE NEEDED - UVA-induced photoreceptor necroptosis paper

Ntentakis, Dimitrios <Dimitrios_Ntentakis@MEEI.HARVARD.EDU>
Tue 11/22/2022 6:29 PM
To: Correa, Victor <Victor_Correa@MEEI.HARVARD.EDU>;YU ZHEN <dryuzhen@hotmail.com>;Efstathiou, Nikolaos <Nikolaos_Efstathiou@meei.harvard.edu>;Henar <henar.albertos@gmail.com>;Kenji Ishihara <ikenx2@kuhp.kyoto-u.ac.jp>;Narimatsu, Toshio <Toshio_Narimatsu@MEEI.HARVARD.EDU>;Vavvas, Demetrios,MD, PhD <Demetrios_Vavvas@meei.harvard.edu>;iesato@shinshu-u.ac.jp <iesato@shinshu-u.ac.jp>;chenxh327@sysu.edu.cn <chenxh327@sysu.edu.cn>

Dear all,

**"I acknowledge the updated author list and accept the changes made to the manuscript"**.

Sincerely,
Dimitrios Ntentakis

---

**From:** Correa, Victor <Victor_Correa@MEEI.HARVARD.EDU>
**Sent:** Tuesday, November 22, 2022 12:08 PM
**To:** YU ZHEN <dryuzhen@hotmail.com>; Correa, Victor <Victor_Correa@MEEI.HARVARD.EDU>; Efstathiou, Nikolaos <Nikolaos_Efstathiou@meei.harvard.edu>; Henar <henar.albertos@gmail.com>; Kenji Ishihara <ikenx2@kuhp.kyoto-u.ac.jp>; Narimatsu, Toshio <Toshio_Narimatsu@MEEI.HARVARD.EDU>; Ntentakis, Dimitrios <Dimitrios_Ntentakis@MEEI.HARVARD.EDU>; Vavvas, Demetrios,MD, PhD <Demetrios_Vavvas@meei.harvard.edu>; iesato@shinshu-u.ac.jp <iesato@shinshu-u.ac.jp>; chenxh327@sysu.edu.cn <chenxh327@sysu.edu.cn>
**Subject:** RESPONSE NEEDED - UVA-induced photoreceptor necroptosis paper

Dear Colleagues,

During the review process of our paper, we have made some changes to the authors list. The updated authors list is as follows:

Zhen Yu*[1,2], Victor S. M. C. Correa*[2], Nikolaos E. Efstathiou[2], Henar Albertos-Arranz[3], Xiaohong Chen[2], Kenji Ishihara[2], Yasuhiro Iesato[2], Toshio Narimatsu[2], Dimitrios Ntentakis[2] and Demetrios G. Vavvas[2]

1 Shenzhen Eye Hospital, Jinan University, Shenzhen Eye Institute, Shenzhen, 518040, China.
2 Retina Service, Ines and Fred Yeatts Retina Research Laboratory, Angiogenesis Laboratory, Department of Ophthalmology, Massachusetts Eye and Ear, Harvard Medical School, Boston, MA, 02114, USA.
3 Department of Physiology, Genetics and Microbiology, University of Alicante, 03690 Alicante, Spain.
*These authors contributed equally to this work

You can access the manuscript submission with the following link: https://mts-cddiscovery.nature.com/cgi-bin/main.plex?el=A2Tf1BTR7D3CDj2F6A9ftdNqr1cRQfxGeKro8lLxoNPgZ

Please reply to acknowledge the updated author list and accepting the changes made to the manuscript
For your convenience, you can just copy the statement: **"I acknowledge the updated author list and accept the changes made to the manuscript"**.
Also, **don't forget to sign the email with your full name.**

Please let me know if you have any questions

Thank you in advance for your expedited response,

Victor San Martin Carvalho Correa, MD
Research Fellow.
Ines and Fred Yeatts Retina Research Laboratory, Mass Eye and Ear.
325 Cambridge Street, 3rd floor, Boston MA

## Re: RESPONSE NEEDED - UVA-induced photoreceptor necroptosis paper

ZHEN YU <dryuzhen@hotmail.com>
Tue 11/22/2022 5:24 PM
To: Henar Albertos <henar.albertos@gmail.com>;Narimatsu, Toshio <Toshio_Narimatsu@MEEI.HARVARD.EDU>
Cc: Correa, Victor <Victor_Correa@MEEI.HARVARD.EDU>;Vavvas, Demetrios,MD, PhD <Demetrios_Vavvas@meei.harvard.edu>;Efstathiou, Nikolaos <Nikolaos_Efstathiou@meei.harvard.edu>;Kenji Ishihara <ikenx2@kuhp.kyoto-u.ac.jp>;Ntentakis, Dimitrios <Dimitrios_Ntentakis@MEEI.HARVARD.EDU>;iesato@shinshu-u.ac.jp <iesato@shinshu-u.ac.jp>;chenxh327@sysu.edu.cn <chenxh327@sysu.edu.cn>

External Email - Use Caution

Hi all!

I acknowledge the updated author list and accept the changes made to the manuscript.

Thank you,
Zhen Yu

获取 Outlook for iOS

---

**发件人:** Henar Albertos <henar.albertos@gmail.com>
**发送时间:** Wednesday, November 23, 2022 1:29:00 AM
**收件人:** Narimatsu, Toshio <Toshio_Narimatsu@meei.harvard.edu>
**抄送:** Correa, Victor <Victor_Correa@meei.harvard.edu>; Vavvas, Demetrios,MD, PhD <Demetrios_Vavvas@meei.harvard.edu>; Zhen Ethan Yu <dryuzhen@hotmail.com>; Efstathiou, Nikolaos <Nikolaos_Efstathiou@meei.harvard.edu>; Kenji Ishihara <ikenx2@kuhp.kyoto-u.ac.jp>; Ntentakis, Dimitrios <Dimitrios_Ntentakis@meei.harvard.edu>; iesato@shinshu-u.ac.jp <iesato@shinshu-u.ac.jp>; chenxh327@sysu.edu.cn <chenxh327@sysu.edu.cn>
**主题:** Re: RESPONSE NEEDED - UVA-induced photoreceptor necroptosis paper

Hi to everyone,

I acknowledge the updated author list and accept the changes made to the manuscript.

Thank you,
Henar Albertos Arranz

El mar, 22 nov 2022 a las 18:26, Narimatsu, Toshio (<Toshio_Narimatsu@meei.harvard.edu>) escribió:

> Hi all,
>
> I acknowledge the updated author list and accept the changes made to the manuscript.
> Thank you.
>
> Toshio Narimatsu
>
> ---
>
> **From:** Vavvas, Demetrios,MD, PhD <Demetrios_Vavvas@meei.harvard.edu>
> **Sent:** Tuesday, November 22, 2022 12:15
> **To:** Correa, Victor <Victor_Correa@MEEI.HARVARD.EDU>
> **Cc:** Zhen Ethan Yu <dryuzhen@hotmail.com>; Efstathiou, Nikolaos <Nikolaos_Efstathiou@meei.harvard.edu>; Henar <henar.albertos@gmail.com>; Kenji Ishihara <ikenx2@kuhp.kyoto-u.ac.jp>; Narimatsu, Toshio <Toshio_Narimatsu@MEEI.HARVARD.EDU>; Ntentakis, Dimitrios <Dimitrios_Ntentakis@MEEI.HARVARD.EDU>; Vavvas, Demetrios,MD, PhD <Demetrios_Vavvas@meei.harvard.edu>; iesato@shinshu-u.ac.jp <iesato@shinshu-u.ac.jp>; chenxh327@sysu.edu.cn <chenxh327@sysu.edu.cn>
> **Subject:** Re: RESPONSE NEEDED - UVA-induced photoreceptor necroptosis paper
>
> YEs agree
>
> **Demetrios G. Vavvas MD, PhD**
> *Solman and Libe Friedman Professor of Ophthalmology*
> Director Retina Service | Harvard Medical School
> Co-Director Ocular Regenerative Medical Institute
> Principal Investigator Ines and Fredrick Yeatts Retinal Research Laboratory
> Mass. Eye and Ear Infirmary and Mass General Hospital
> 243 Charles St. Boston MA 02114
> **P** 617-573-6874 **E:** vavvas@meei.harvard.edu
>
> > On Nov 22, 2022, at 12:08 PM, Correa, Victor <Victor_Correa@MEEI.HARVARD.EDU> wrote:
> >
> > Dear Colleagues,
> >
> > During the review process of our paper, we have made some changes to the authors list. The updated authors list is as follows:

Zhen Yu*[1,2], Victor S. M. C. Correa*[2], Nikolaos E. Efstathiou[2], Henar Albertos-Arranz[3], Xiaohong Chen[2], Kenji Ishihara[2], Yasuhiro Iesato[2], Toshio Narimatsu[2], Dimitrios Ntentakis[2] and Demetrios G. Vavvas[2]
1 Shenzhen Eye Hospital, Jinan University, Shenzhen Eye Institute, Shenzhen, 518040, China.
2 Retina Service, Ines and Fred Yeatts Retina Research Laboratory, Angiogenesis Laboratory, Department of Ophthalmology, Massachusetts Eye and Ear, Harvard Medical School, Boston, MA, 02114, USA.
3 Department of Physiology, Genetics and Microbiology, University of Alicante, 03690 Alicante, Spain.
*These authors contributed equally to this work

You can access the manuscript submission with the following link: https://mts-cddiscovery.nature.com/cgi-bin/main.plex?el=A2Tf1BTR7D3CDj2F6A9ftdNqr1cRQfxGeKro8lLxoNPgZ

Please reply to acknowledge the updated author list and accepting the changes made to the manuscript
For your convenience, you can just copy the statement: **"I acknowledge the updated author list and accept the changes made to the manuscript"**.
Also, **don't forget to sign the email with your full name.**

Please let me know if you have any questions

Thank you in advance for your expedited response,

Victor San Martin Carvalho Correa, MD
Research Fellow.
Ines and Fred Yeatts Retina Research Laboratory, Mass Eye and Ear.
325 Cambridge Street, 3rd floor, Boston MA

The information in this e-mail is intended only for the person to whom it is addressed. If you believe this e-mail was sent to you in error and the e-mail contains patient information, please contact the Mass General Brigham Compliance HelpLine at https://www.massgeneralbrigham.org/complianceline .

--
**Henar Albertos-Arranz, Msc.**
Investigadora predoctoral FPU
*Neurobiología del Sistema Visual y terapia de enfermedades neurodegenerativas (NEUROVIS)*
Dpto. Fisiología, Genética y Microbiología
Universidad de Alicante
(Pabellón 13, ext. 1194; +34 630 306 072 1194)

**Re: RESPONSE NEEDED - UVA-induced photoreceptor necroptosis paper**

Kenji Ishihara <ikenx2@kuhp.kyoto-u.ac.jp>
Tue 11/22/2022 6:35 PM

To: Ntentakis, Dimitrios <Dimitrios_Ntentakis@MEEI.HARVARD.EDU>

Cc: Correa, Victor <Victor_Correa@MEEI.HARVARD.EDU>;Efstathiou, Nikolaos <Nikolaos_Efstathiou@meei.harvard.edu>;Henar <henar.albertos@gmail.com>;Narimatsu, Toshio <Toshio_Narimatsu@MEEI.HARVARD.EDU>;Vavvas, Demetrios,MD, PhD <Demetrios_Vavvas@meei.harvard.edu>;YU ZHEN <dryuzhen@hotmail.com>;chenxh327@sysu.edu.cn <chenxh327@sysu.edu.cn>;iesato@shinshu-u.ac.jp <iesato@shinshu-u.ac.jp>

External Email - Use Caution

Hi all.

I acknowledge the updated author list and accept the changes made to the manuscript.

Kenji Ishihara

2022年11月23日(水) 8:30 Ntentakis, Dimitrios <Dimitrios_Ntentakis@meei.harvard.edu>:

> Dear all,
>
> **"I acknowledge the updated author list and accept the changes made to the manuscript"**.
>
> Sincerely,
> Dimitrios Ntentakis
>
> ---
>
> **From:** Correa, Victor <Victor_Correa@MEEI.HARVARD.EDU>
> **Sent:** Tuesday, November 22, 2022 12:08 PM
> **To:** YU ZHEN <dryuzhen@hotmail.com>; Correa, Victor <Victor_Correa@MEEI.HARVARD.EDU>; Efstathiou, Nikolaos <Nikolaos_Efstathiou@meei.harvard.edu>; Henar <henar.albertos@gmail.com>; Kenji Ishihara <ikenx2@kuhp.kyoto-u.ac.jp>; Narimatsu, Toshio <Toshio_Narimatsu@MEEI.HARVARD.EDU>; Ntentakis, Dimitrios <Dimitrios_Ntentakis@MEEI.HARVARD.EDU>; Vavvas, Demetrios,MD, PhD <Demetrios_Vavvas@meei.harvard.edu>; iesato@shinshu-u.ac.jp <iesato@shinshu-u.ac.jp>; chenxh327@sysu.edu.cn <chenxh327@sysu.edu.cn>
> **Subject:** RESPONSE NEEDED - UVA-induced photoreceptor necroptosis paper
>
> Dear Colleagues,
>
> During the review process of our paper, we have made some changes to the authors list. The updated authors list is as follows:
>
> Zhen Yu*[1,2], Victor S. M. C. Correa*[2], Nikolaos E. Efstathiou[2], Henar Albertos-Arranz[3], Xiaohong Chen[2], Kenji Ishihara[2], Yasuhiro Iesato[2], Toshio Narimatsu[2], Dimitrios Ntentakis[2] and Demetrios G. Vavvas[2]
>
> 1 Shenzhen Eye Hospital, Jinan University, Shenzhen Eye Institute, Shenzhen, 518040, China.
>
> 2 Retina Service, Ines and Fred Yeatts Retina Research Laboratory, Angiogenesis Laboratory, Department of Ophthalmology, Massachusetts Eye and Ear, Harvard Medical School, Boston, MA, 02114, USA.
>
> 3 Department of Physiology, Genetics and Microbiology, University of Alicante, 03690 Alicante, Spain.
>
> *These authors contributed equally to this work
>
> You can access the manuscript submission with the following link: https://mts-cddiscovery.nature.com/cgi-bin/main.plex?el=A2Tf1BTR7D3CDj2F6A9ftdNqr1cRQfxGeKro8lLxoNPgZ
>
> Please reply to acknowledge the updated author list and accepting the changes made to the manuscript
> For your convenience, you can just copy the statement: **"I acknowledge the updated author list and accept the changes made to the manuscript"**.
> Also, **don't forget to sign the email with your full name.**
>
> Please let me know if you have any questions
>
> Thank you in advance for your expedited response,
>
> Victor San Martin Carvalho Correa, MD
> Research Fellow.
> Ines and Fred Yeatts Retina Research Laboratory, Mass Eye and Ear.

325 Cambridge Street, 3rd floor, Boston MA

The information in this e-mail is intended only for the person to whom it is addressed. If you believe this e-mail was sent to you in error and the e-mail contains patient information, please contact the Mass General Brigham Compliance HelpLine at https://www.massgeneralbrigham.org/complianceline .

Please note that this e-mail is not secure (encrypted). If you do not wish to continue communication over unencrypted e-mail, please notify the sender of this message immediately. Continuing to send or respond to e-mail after receiving this message means you understand and accept this risk and wish to continue to communicate over unencrypted e-mail.

**Re: RESPONSE NEEDED - UVA-induced photoreceptor necroptosis paper**

陈晓虹 <chenxh327@mail.sysu.edu.cn>
Tue 11/22/2022 9:32 PM

To: Correa, Victor <Victor_Correa@MEEI.HARVARD.EDU>

Cc: YU ZHEN <dryuzhen@hotmail.com>;Efstathiou, Nikolaos <Nikolaos_Efstathiou@meei.harvard.edu>;Henar <henar.albertos@gmail.com>;Kenji Ishihara <ikenx2@kuhp.kyoto-u.ac.jp>;Narimatsu, Toshio <Toshio_Narimatsu@MEEI.HARVARD.EDU>;Ntentakis, Dimitrios <Dimitrios_Ntentakis@MEEI.HARVARD.EDU>;Vavvas, Demetrios,MD, PhD <Demetrios_Vavvas@meei.harvard.edu>;iesato@shinshu-u.ac.jp <iesato@shinshu-u.ac.jp>

External Email - Use Caution

Dear all

I acknowledge the updated author list and accept the changes made to the manuscript

Sincerely,
Xiaohong Chen

-----原始邮件-----
**发件人:**"Correa, Victor" <Victor_Correa@MEEI.HARVARD.EDU>
**发送时间:**2022-11-23 01:08:31 (星期三)
**收件人:** "YU ZHEN" <dryuzhen@hotmail.com>, "Correa, Victor" <Victor_Correa@MEEI.HARVARD.EDU>, "Efstathiou, Nikolaos" <Nikolaos_Efstathiou@meei.harvard.edu>, Henar <henar.albertos@gmail.com>, "Kenji Ishihara" <ikenx2@kuhp.kyoto-u.ac.jp>, "Narimatsu, Toshio" <Toshio_Narimatsu@MEEI.HARVARD.EDU>, "Ntentakis, Dimitrios" <Dimitrios_Ntentakis@MEEI.HARVARD.EDU>, "Vavvas, Demetrios,MD, PhD" <Demetrios_Vavvas@meei.harvard.edu>, "iesato@shinshu-u.ac.jp" <iesato@shinshu-u.ac.jp>, "chenxh327@sysu.edu.cn" <chenxh327@sysu.edu.cn>
**抄送:**
**主题:** RESPONSE NEEDED - UVA-induced photoreceptor necroptosis paper

Dear Colleagues,

During the review process of our paper, we have made some changes to the authors list. The updated authors list is as follows:

Zhen Yu*[1,2], Victor S. M. C. Correa*[2], Nikolaos E. Efstathiou[2], Henar Albertos-Arranz[3], Xiaohong Chen[2], Kenji Ishihara[2], Yasuhiro Iesato[2], Toshio Narimatsu[2], Dimitrios Ntentakis[2] and Demetrios G. Vavvas[2]

1 Shenzhen Eye Hospital, Jinan University, Shenzhen Eye Institute, Shenzhen, 518040, China.
2 Retina Service, Ines and Fred Yeatts Retina Research Laboratory, Angiogenesis Laboratory, Department of Ophthalmology, Massachusetts Eye and Ear, Harvard Medical School, Boston, MA, 02114, USA.
3 Department of Physiology, Genetics and Microbiology, University of Alicante, 03690 Alicante, Spain.
*These authors contributed equally to this work

You can access the manuscript submission with the following link: https://mts-cddiscovery.nature.com/cgi-bin/main.plex?el=A2Tf1BTR7D3CDj2F6A9ftdNqr1cRQfxGeKro8lLxoNPgZ

Please reply to acknowledge the updated author list and accepting the changes made to the manuscript
For your convenience, you can just copy the statement: **"I acknowledge the updated author list and accept the changes made to the manuscript"**.
Also, **don't forget to sign the email with your full name.**

Please let me know if you have any questions

Thank you in advance for your expedited response,

Victor San Martin Carvalho Correa, MD
Research Fellow.
Ines and Fred Yeatts Retina Research Laboratory, Mass Eye and Ear.
325 Cambridge Street, 3rd floor, Boston MA

The information in this e-mail is intended only for the person to whom it is addressed. If you believe this e-mail was sent to you in error and the e-mail contains patient information, please contact the Mass General Brigham Compliance HelpLine at https://www.massgeneralbrigham.org/complianceline .

Please note that this e-mail is not secure (encrypted). If you do not wish to continue communication over unencrypted e-mail, please notify the sender of this message immediately. Continuing to send or respond to e-mail after receiving this message means you understand and accept this risk and wish to continue to communicate over unencrypted e-mail.

本邮件及其附件含有发送给特定个人和用于特定目的的信息。如果您不是预期的收件人，请立即删除本邮件并通知发件人。严禁任何非预期的收件人使用、传播、分发或复制本邮件或其附件。
This email and its attachments may contain confidential information intended for a specific individual and purpose. If you are not the

intended recipient, you should delete this email and notify the sender immediately. Any use, dissemination, distribution, or copying of this email or its attachments by persons other than the intended recipient(s), is strictly prohibited.

**Re: RESPONSE NEEDED - UVA-induced photoreceptor necroptosis paper**

家里康弘 <iesato@shinshu-u.ac.jp>
Wed 11/23/2022 4:03 AM
To: Correa, Victor <Victor_Correa@MEEI.HARVARD.EDU>;陈晓虹 <chenxh327@mail.sysu.edu.cn>
Cc: YU ZHEN <dryuzhen@hotmail.com>;Efstathiou, Nikolaos <Nikolaos_Efstathiou@meei.harvard.edu>;Henar <henar.albertos@gmail.com>;Kenji Ishihara <ikenx2@kuhp.kyoto-u.ac.jp>;Narimatsu, Toshio <Toshio_Narimatsu@MEEI.HARVARD.EDU>;Ntentakis, Dimitrios <Dimitrios_Ntentakis@MEEI.HARVARD.EDU>;Vavvas, Demetrios,MD, PhD <Demetrios_Vavvas@meei.harvard.edu>

External Email - Use Caution

Dear all,

I acknowledge the updated author list and accept the changes made to the manuscript.

Sincerely,
Yasuhiro Iesato
2022年11月23日 AM11:32 +0900、陈晓虹 <chenxh327@mail.sysu.edu.cn>のメール:

> Dear all
>
> I acknowledge the updated author list and accept the changes made to the manuscript
>
> Sincerely,
> Xiaohong Chen
>
> > -----原始邮件-----
> > **发件人:**"Correa, Victor" <Victor_Correa@MEEI.HARVARD.EDU>
> > **发送时间:**2022-11-23 01:08:31 (星期三)
> > **收件人:** "YU ZHEN" <dryuzhen@hotmail.com>, "Correa, Victor" <Victor_Correa@MEEI.HARVARD.EDU>, "Efstathiou, Nikolaos" <Nikolaos_Efstathiou@meei.harvard.edu>, Henar <henar.albertos@gmail.com>, "Kenji Ishihara" <ikenx2@kuhp.kyoto-u.ac.jp>, "Narimatsu, Toshio" <Toshio_Narimatsu@MEEI.HARVARD.EDU>, "Ntentakis, Dimitrios" <Dimitrios_Ntentakis@MEEI.HARVARD.EDU>, "Vavvas, Demetrios,MD, PhD" <Demetrios_Vavvas@meei.harvard.edu>, "iesato@shinshu-u.ac.jp" <iesato@shinshu-u.ac.jp>, "chenxh327@sysu.edu.cn" <chenxh327@sysu.edu.cn>
> > **抄送:**
> > **主题:** RESPONSE NEEDED - UVA-induced photoreceptor necroptosis paper
> >
> > Dear Colleagues,
> >
> > During the review process of our paper, we have made some changes to the authors list. The updated authors list is as follows:
> >
> > Zhen Yu*[1,2], Victor S. M. C. Correa*[2], Nikolaos E. Efstathiou[2], Henar Albertos-Arranz[3], Xiaohong Chen[2], Kenji Ishihara[2], Yasuhiro Iesato[2], Toshio Narimatsu[2], Dimitrios Ntentakis[2] and Demetrios G. Vavvas[2]
> >
> > 1 Shenzhen Eye Hospital, Jinan University, Shenzhen Eye Institute, Shenzhen, 518040, China.
> > 2 Retina Service, Ines and Fred Yeatts Retina Research Laboratory, Angiogenesis Laboratory, Department of Ophthalmology, Massachusetts Eye and Ear, Harvard Medical School, Boston, MA, 02114, USA.
> > 3 Department of Physiology, Genetics and Microbiology, University of Alicante, 03690 Alicante, Spain.
> > *These authors contributed equally to this work
> >
> > You can access the manuscript submission with the following link: https://mts-cddiscovery.nature.com/cgi-bin/main.plex?el=A2Tf1BTR7D3CDj2F6A9ftdNqr1cRQfxGeKro8lLxoNPgZ
> >
> > Please reply to acknowledge the updated author list and accepting the changes made to the manuscript
> > For your convenience, you can just copy the statement: **"I acknowledge the updated author list and accept the changes made to the manuscript"**.
> > Also, **don't forget to sign the email with your full name.**
> >
> > Please let me know if you have any questions
> >
> > Thank you in advance for your expedited response,
> >
> > Victor San Martin Carvalho Correa, MD
> > Research Fellow.
> > Ines and Fred Yeatts Retina Research Laboratory, Mass Eye and Ear.
> > 325 Cambridge Street, 3rd floor, Boston MA
> > The information in this e-mail is intended only for the person to whom it is addressed. If you believe this e-mail was sent to you in error and the e-mail contains patient information, please contact the Mass General Brigham Compliance HelpLine at https://www.massgeneralbrigham.org/complianceline .
> >
> > Please note that this e-mail is not secure (encrypted). If you do not wish to continue communication over unencrypted e-mail, please notify the sender of this message immediately. Continuing to send or respond to e-mail after receiving this message means you understand and accept this risk and wish to continue to communicate over unencrypted e-mail.

本邮件及其附件含有发送给特定个人和用于特定目的的信息。如果您不是预期的收件人，请立即删除本邮件并通知发件人。严禁任何非预期的收件人使用、传播、分发或复制本邮件或其附件。

This email and its attachments may contain confidential information intended for a specific individual and purpose. If you are not the intended recipient, you should delete this email and notify the sender immediately. Any use, dissemination, distribution, or copying of this email or its attachments by persons other than the intended recipient(s), is strictly prohibited.

**Re: RESPONSE NEEDED - UVA-induced photoreceptor necroptosis paper**

Correa, Victor <Victor_Correa@MEEI.HARVARD.EDU>
Wed 11/23/2022 9:12 AM
To: 家里康弘 <iesato@shinshu-u.ac.jp>;陈晓虹 <chenxh327@mail.sysu.edu.cn>
Cc: YU ZHEN <dryuzhen@hotmail.com>;Efstathiou, Nikolaos <Nikolaos_Efstathiou@meei.harvard.edu>;Henar <henar.albertos@gmail.com>;Kenji Ishihara <ikenx2@kuhp.kyoto-u.ac.jp>;Narimatsu, Toshio <Toshio_Narimatsu@MEEI.HARVARD.EDU>;Ntentakis, Dimitrios <Dimitrios_Ntentakis@MEEI.HARVARD.EDU>;Vavvas, Demetrios,MD, PhD <Demetrios_Vavvas@meei.harvard.edu>

Dear all,

I acknowledge the updated author list and accept the changes made to the manuscript.

Sincerely,

Victor San Martin Carvalho Correa

---

**From:** 家里康弘 <iesato@shinshu-u.ac.jp>
**Sent:** Wednesday, November 23, 2022 4:03 AM
**To:** Correa, Victor <Victor_Correa@MEEI.HARVARD.EDU>; 陈晓虹 <chenxh327@mail.sysu.edu.cn>
**Cc:** YU ZHEN <dryuzhen@hotmail.com>; Efstathiou, Nikolaos <Nikolaos_Efstathiou@meei.harvard.edu>; Henar <henar.albertos@gmail.com>; Kenji Ishihara <ikenx2@kuhp.kyoto-u.ac.jp>; Narimatsu, Toshio <Toshio_Narimatsu@MEEI.HARVARD.EDU>; Ntentakis, Dimitrios <Dimitrios_Ntentakis@MEEI.HARVARD.EDU>; Vavvas, Demetrios,MD, PhD <Demetrios_Vavvas@meei.harvard.edu>
**Subject:** Re: RESPONSE NEEDED - UVA-induced photoreceptor necroptosis paper

External Email - Use Caution

Dear all,

I acknowledge the updated author list and accept the changes made to the manuscript.

Sincerely,
Yasuhiro Iesato
2022年11月23日 AM11:32 +0900、陈晓虹 <chenxh327@mail.sysu.edu.cn>のメール:

> Dear all
>
> I acknowledge the updated author list and accept the changes made to the manuscript
>
> Sincerely,
> Xiaohong Chen
>
>> -----原始邮件-----
>> **发件人:**"Correa, Victor" <Victor_Correa@MEEI.HARVARD.EDU>
>> **发送时间:**2022-11-23 01:08:31 (星期三)
>> **收件人:** "YU ZHEN" <dryuzhen@hotmail.com>, "Correa, Victor" <Victor_Correa@MEEI.HARVARD.EDU>, "Efstathiou, Nikolaos" <Nikolaos_Efstathiou@meei.harvard.edu>, Henar <henar.albertos@gmail.com>, "Kenji Ishihara" <ikenx2@kuhp.kyoto-u.ac.jp>, "Narimatsu, Toshio" <Toshio_Narimatsu@MEEI.HARVARD.EDU>, "Ntentakis, Dimitrios" <Dimitrios_Ntentakis@MEEI.HARVARD.EDU>, "Vavvas, Demetrios,MD, PhD" <Demetrios_Vavvas@meei.harvard.edu>, "iesato@shinshu-u.ac.jp" <iesato@shinshu-u.ac.jp>, "chenxh327@sysu.edu.cn" <chenxh327@sysu.edu.cn>
>> **抄送:**
>> **主题:** RESPONSE NEEDED - UVA-induced photoreceptor necroptosis paper
>>
>> Dear Colleagues,
>>
>> During the review process of our paper, we have made some changes to the authors list. The updated authors list is as follows:
>>
>> Zhen Yu*[1,2], Victor S. M. C. Correa*[2], Nikolaos E. Efstathiou[2], Henar Albertos-Arranz[3], Xiaohong Chen[2], Kenji Ishihara[2], Yasuhiro Iesato[2], Toshio Narimatsu[2], Dimitrios Ntentakis[2] and Demetrios G. Vavvas[2]
>>
>> 1 Shenzhen Eye Hospital, Jinan University, Shenzhen Eye Institute, Shenzhen, 518040, China.
>> 2 Retina Service, Ines and Fred Yeatts Retina Research Laboratory, Angiogenesis Laboratory, Department of Ophthalmology, Massachusetts Eye and Ear, Harvard Medical School, Boston, MA, 02114, USA.
>> 3 Department of Physiology, Genetics and Microbiology, University of Alicante, 03690 Alicante, Spain.
>> *These authors contributed equally to this work
>>
>> You can access the manuscript submission with the following link: https://mts-cddiscovery.nature.com/cgi-bin/main.plex?el=A2Tf1BTR7D3CDj2F6A9ftdNqr1cRQfxGeKro8lLxoNPgZ
>>
>> Please reply to acknowledge the updated author list and accepting the changes made to the manuscript
>> For your convenience, you can just copy the statement: **"I acknowledge the updated author list and accept the changes made to the manuscript"**.
>> Also, **don't forget to sign the email with your full name.**

Please let me know if you have any questions

Thank you in advance for your expedited response,

Victor San Martin Carvalho Correa, MD
Research Fellow.
Ines and Fred Yeatts Retina Research Laboratory, Mass Eye and Ear.
325 Cambridge Street, 3rd floor, Boston MA

The information in this e-mail is intended only for the person to whom it is addressed. If you believe this e-mail was sent to you in error and the e-mail contains patient information, please contact the Mass General Brigham Compliance HelpLine at https://www.massgeneralbrigham.org/complianceline .

Please note that this e-mail is not secure (encrypted). If you do not wish to continue communication over unencrypted e-mail, please notify the sender of this message immediately. Continuing to send or respond to e-mail after receiving this message means you understand and accept this risk and wish to continue to communicate over unencrypted e-mail.

---

本邮件及其附件含有发送给特定个人和用于特定目的的信息。如果您不是预期的收件人，请立即删除本邮件并通知发件人。严禁任何非预期的收件人使用、传播、分发或复制本邮件或其附件。

This email and its attachments may contain confidential information intended for a specific individual and purpose. If you are not the intended recipient, you should delete this email and notify the sender immediately. Any use, dissemination, distribution, or copying of this email or its attachments by persons other than the intended recipient(s), is strictly prohibited.
