# Supplementary figures and images for: UVA induces retinal photoreceptor cell death via receptor interacting protein 3 kinase mediated necroptosis
Source: Cell Death Discov. 2022 Dec 12;8:489. doi: 10.1038/s41420-022-01273-1 (PMC9744841; doi:10.1038/s41420-022-01273-1)

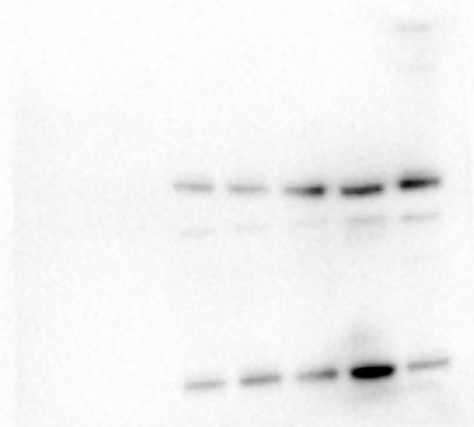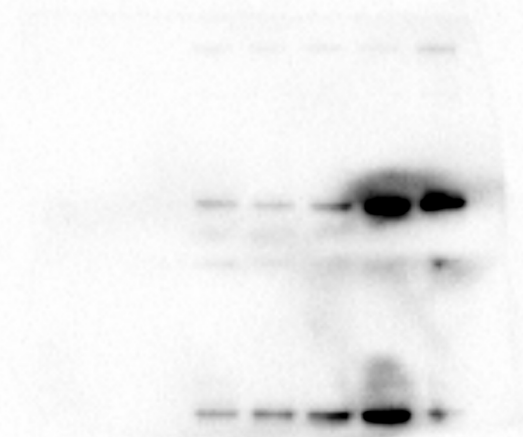

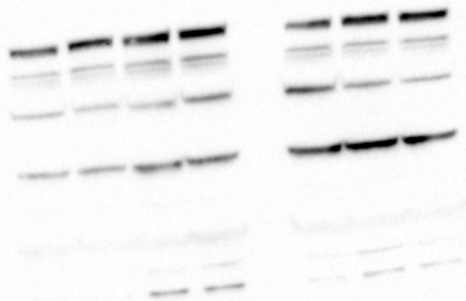

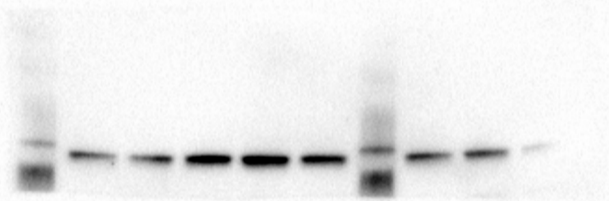

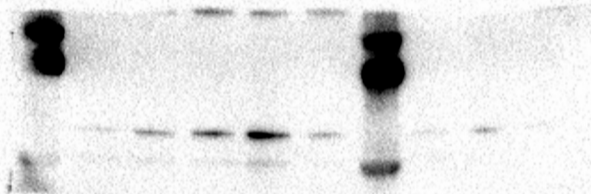

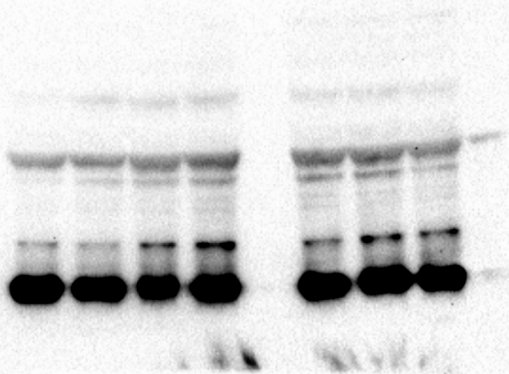

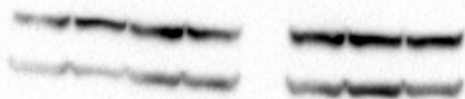

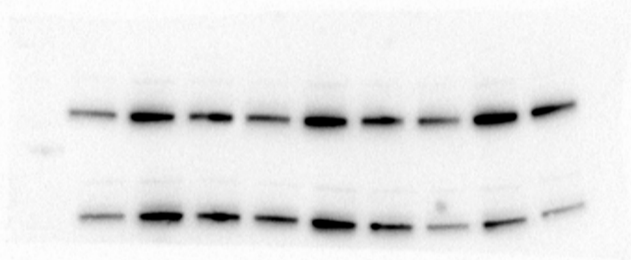

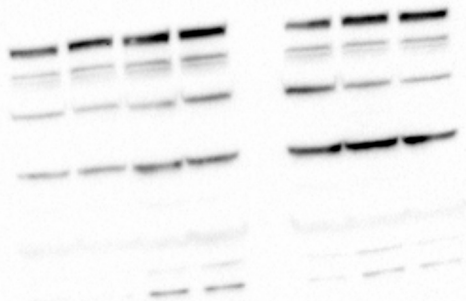

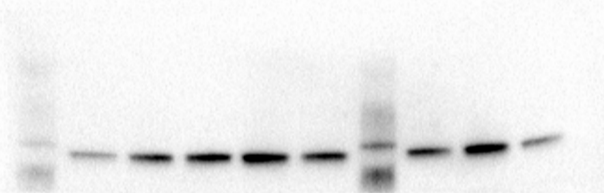

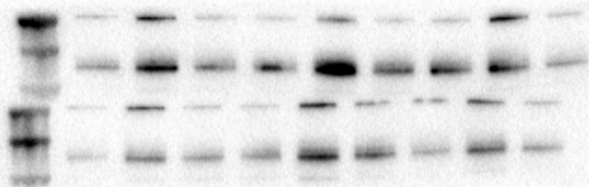

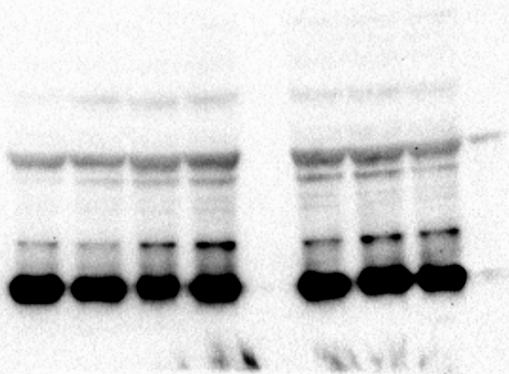

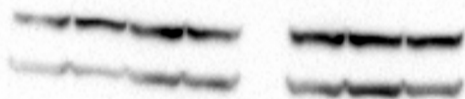

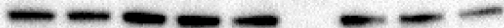

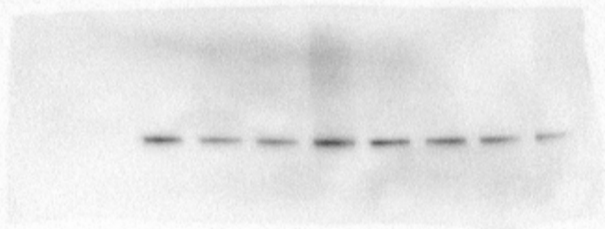

Supplement: Supplementary file 3 — Original western blots [file 41420_2022_1273_MOESM3_ESM.pdf]

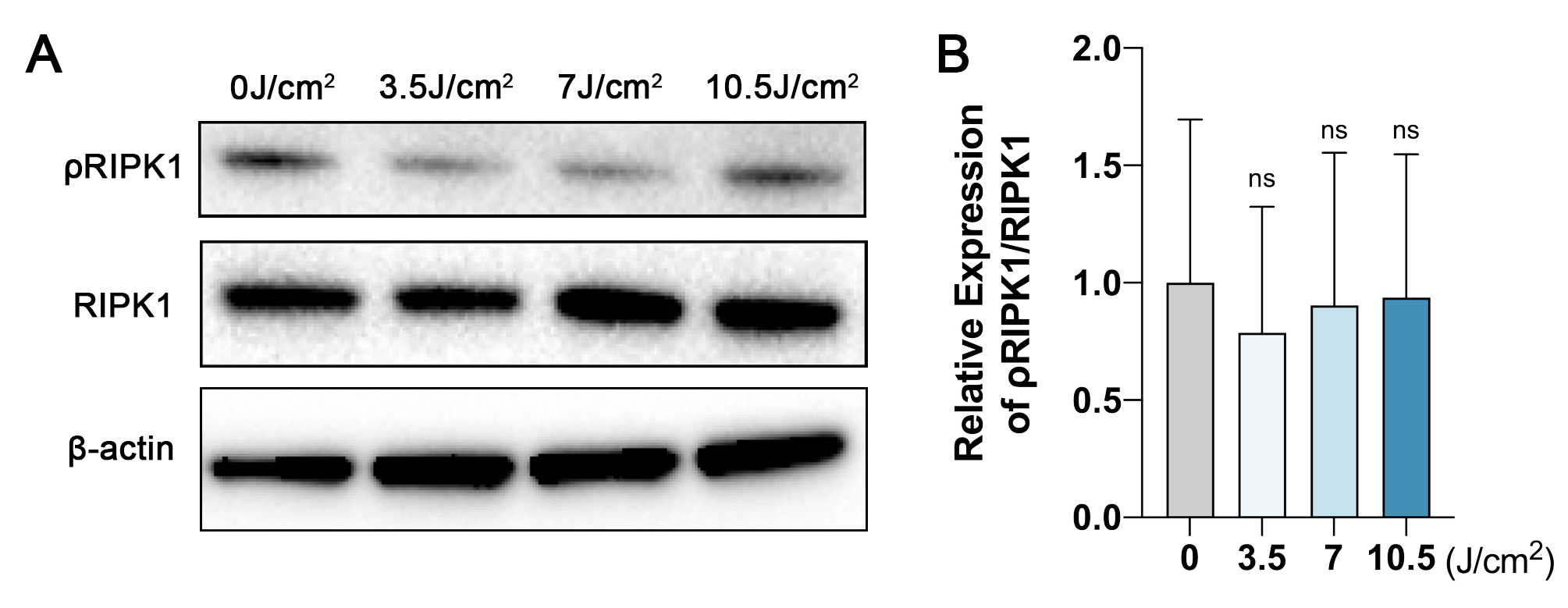

Supplement: Supplementary file 4 — Figure S1 [file 41420_2022_1273_MOESM4_ESM.tif]
