# Supplementary material for: UVA induces retinal photoreceptor cell death via receptor interacting protein 3 kinase mediated necroptosis
Source: Cell Death Discov. 2022 Dec 12;8:489. doi: 10.1038/s41420-022-01273-1 (PMC9744841; doi:10.1038/s41420-022-01273-1)
Supplement: Supplementary file 5 — Table S1 [file 41420_2022_1273_MOESM5_ESM.docx]

**Table S1**. Antibodies for immunofluorescence and western blot used in this study.

| Antigen | Company | Catalog # | Dilution (IF) | Dilution (WB) |
| --- | --- | --- | --- | --- |
| RIP3 | NOVUS | NBP1-77299 |  | 1:1 000 |
| RIP3 | SIGMA | R4277 | 1:200 |  |
| ρ-RIP3 | ABCAM | ab195117 |  | 1:3000 |
| ρ-RIP1 | Thermo Fisher | PA5-105640 |  | 1:1 000 |
| RIP1 | BD Biosciences | 610459 |  | 1:1 000 |
| MLKL | Biorbyt | orb32399 |  | 1:800 |
| PARP | GeneTex | GTX100573 |  | 1:800 |
| Cleaved Caspase-8 | Enzo | AXL-804-447 |  | 1:1000 |
| Cleaved  Caspase-3 | CST | 9661 |  | 1:1000 |
| 4HNE | ABCAM | ab46544 | 1:100 |  |
| β-ACTIN | CST | 4970S |  | 1:1 000 |
